# Synthesis and Solvent Dependent Fluorescence of Some Piperidine-Substituted Naphthalimide Derivatives and Consequences for Water Sensing

**DOI:** 10.3390/ijms23052760

**Published:** 2022-03-02

**Authors:** Radu Tigoianu, Anton Airinei, Emilian Georgescu, Alina Nicolescu, Florentina Georgescu, Dragos Lucian Isac, Calin Deleanu, Florin Oancea

**Affiliations:** 1 Petru Poni Institute of Macromolecular Chemistry, Romanian Academy, Aleea Grigore Ghica Voda 41A, 700487 Iasi, Romania; tigoianu.radu@icmpp.ro (R.T.); isac.dragos@icmpp.ro (D.L.I.); calind@icmpp.ro (C.D.); 2 C. D. Nenitescu Centre of Organic Chemistry, Romanian Academy, Splaiul Independentei 202B, 060023 Bucharest, Romania; g_emilian@yahoo.com; 3 Research Center, Chimcomplex S.A., St. Uzinei 1, 240050 Ramnicu Valcea, Romania; 4 Enpro Soctech Com Srl, Str. Elefterie 51, 050524 Bucharest, Romania; florentina_fg@yahoo.com; 5 National Research and Development Institute for Chemistry and Petrochemistry—ICECHIM, Splaiul Independentei 202B, 060023 Bucharest, Romania; florin.oancea@icechim.ro

**Keywords:** piperidine-1,8-naphthalimide, solvatochromism, solvent dependent emission, water sensor, fluorescence lifetime

## Abstract

Novel fluorescent strigolactone derivatives that contain the piperidine-substituted 1,8-naphthalimide ring system connected through an ether link to a bioactive 3-methyl-furan-2-one unit were synthesized and their spectroscopic properties investigated. The solvatochromic behavior of these piperidine-naphthalimides was monitored in solvents of different polarity using the electronic absorption and fluorescence spectra. These compounds exhibited a strong positive solvatochromism taking into account the change of solvent polarity, and the response mechanism was analyzed by fluorescence lifetime measurements. According to Catalan and [f(n), f(ε), β, α] solvent scales, the dipolarity and polarizability are relevant to describe the solute–solvent interactions. The emission chemosensing activity was discussed in order to determine the water content in organic environments. The emission intensity of these compounds decreased rapidly in dioxane, increasing water level up to 10%. Measuring of quantum yield indicated that the highest values of quantum efficiency were obtained in nonpolar solvents, while in polar solvents these derivatives revealed the lowest quantum yield. The fluorescence decay can be described by a monoexponential model for low water levels, and for higher water contents a biexponential model was valid.

## 1. Introduction

Naphthalimide derivatives are of interest due to their promising properties such as strong emission, high quantum efficiency, good photostability, thermal stability, film-forming ability, etc. [1,2,3,4,5]. These versatile characteristics make naphthalimides potential materials for fluorescent sensors and molecular switchers, laser active media, light emitting diodes, dyes for natural synthetic fibers, fluorescent markers in biology, antitumor treatments, DNA targeting binders, and photoinitiators [2,3,6,7,8,9,10,11,12,13,14,15,16]. The optical and photophysical properties of naphthalimide derivatives depend on the substituent nature of the heterocycle as well as the position of the substituents [2,17]. The chemical modification of the naphthalimide structure mainly on the imide N site or at the position 4 of the naphthalene moiety gives the possibility to tune their absorption and emission parameters. By substituting electron-donating groups at the C-4 position of the naphthalimides, the emission quantum yield can be usually increased and the fluorescence color can be varied from blue to yellowish-green [13,15,18,19]. Also, the photophysical properties of the naphthalimide derivatives can be tuned by the appropriate choice of the push-pull substituents at imide nitrogen and C4 position at naphthalene ring due to the intramolecular charge transfer in this system [18,20,21].

Naphthalimide derivatives are very attractive to be used as photoactive components in colorimetric and fluorometric chemosensors due to the great diversity of their photophysical characteristics. Therefore, the naphthalimide compounds have been applied to detect different metal ions such as Cu^2+^, Zn^2+^, Hg^2+^, Ag^+^, Fe^3+^, Cr^3+^, or fluoride ion [13,22,23,24,25]. Also, naphthalimide derivatives were explored successfully for monitoring hypochlorous acid and hypochlorite [26,27,28]. Some naphthalimide-based fluorescent compounds were developed for detecting hydrogen sulfide, formaldehyde, or phosgene [29,30,31,32].

The absorption and fluorescence spectra of naphthalimides are sensitive to the polarity of the surrounding environment and some solvent mediated spectral investigations were performed until now [4,17,20,33,34]. The solvatochromic features of the naphthalimide derivatives made them excellent candidates for fluorescent sensors for water detection in solution because the emission is strongly dependent on the solvent polarity and it is quenched even at low water levels [35,36,37]. Also, significant solvatochromism of the emission was encountered in novel naphthalimide derivatives utilized for fluorescence sensing of dimethylformamide (DMF), dimethyl sulfoxide (DMSO) in water, blood plasma, and human urine; or for detection of dichloromethane and 1,4-dioxane [38,39].

Previously, we reported a new compound bearing a 1,8-naphthalimide ring, namely 2-(4-methyl-5-oxo-2,5-dihydro-furan-2-yloxy)-benzo[*de*]isoquinoline-1,3-dione, that proved to be active as a signaling molecule in the rhizosphere [40]. This bioactive molecule is part of a large group of synthetic compounds that mimic the different biological activities of naturally occurring strigolactones. Naturally, strigolactones are plant secondary metabolites that act as both exo-signals for the germination of parasitic plant seeds [41,42], for the stimulation of hyphal branching in arbuscular mycorrhizal fungi (AMF) [43,44], and as phytohormonal endo-signals controlling the architecture of various plant organs [45,46]. All these data prompted us to conceive new potentially bioactive and fluorescent compounds that mimic the various biological activities of naturally strigolactones (SL mimics), suitable for bio-imaging studies in plant cells based on piperidine-substituted 1,8-naphthalimide ring systems connected by an ether link to a bioactive 3-methyl-furan-2-one unit. Here, the solvatochromic behavior of two newly synthesized piperidine-naphthalimide derivatives in several solvents as well as their water detection ability in organic solvents were analyzed. The water-induced fluorescent responses in organic solvents were investigated based on fluorescence quantum yields and decay lifetimes determination. Also, solid state emission of these naphthalimides was evidenced.

## 2. Results and Discussion

**Scheme 1:**
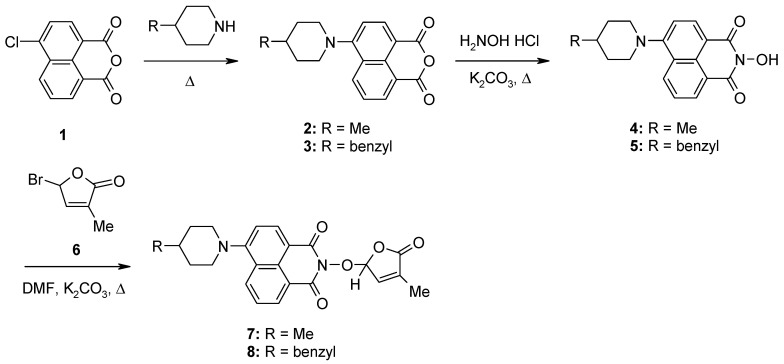
Synthetic procedure for SL mimics **7** and **8**.

The synthetic procedure for the preparation of the two potentially fluorescent SL derivatives containing the piperidine-substituted 1,8-naphthalimide ring system linked through an ether group to a bioactive 3-methyl-furan-2-one moiety started from the commercially available 4-chloro-1,8-naphthalic anhydride (6-chlorobenzo[*de*]isochromene-1,3-dione) **1**. Therefore, by the reactions of 4-chloro-1,8-naphthalic anhydride **1** with some cyclic secondary amines such as 4-methylpiperidine and 4-benzylpiperidine, respectively, 4-amino-1,8-naphthalic anhydride derivatives **2** and **3** were obtained. In the next step, the resulting 4-amino-1,8-naphthalic anhydride compounds **2** and **3** reacted with hydroxylamine hydrochloride in dioxane, under basic conditions, at reflux temperature, to give 2-hydroxy-6-amino-1,8-naphthalimide intermediates **4** and **5**, respectively [47]. These intermediates presented interesting photophysical properties that convinced us that they are useful fluorophores for the syntheses of potentially fluorescent naphthalimide-based strigolactone mimics [47]. Consequently, by the coupling reactions of 2-hydroxy-6-amino-1,8-naphthalimide intermediates **4** and **5** with 5-bromo-3-methyl-5*H*-furan-2-one **6**, we accessed new potentially fluorescent derivatives **7** and **8**, respectively. The reaction was carried out in dimethylformamide (DMF), in the presence of anhydrous potassium carbonate, as basic catalyst, at reflux temperature (Scheme 1).

**Figure 1:**
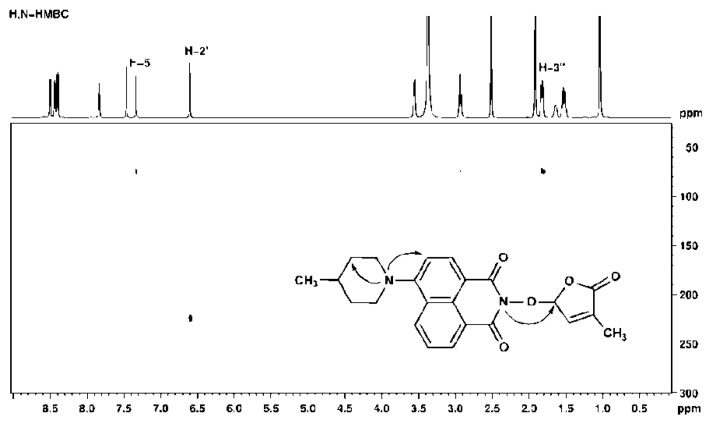
^1^H-^15^N HMBC spectrum for compound **7** recorded in DMSO-d6 at 600 MHz.

The structures of newly synthesized SL mimics have been confirmed based on the information obtained from ^1^H, ^13^C, and ^15^N NMR experiments. The number of proton and carbon NMR signals, chemical shift values, and coupling patterns correspond to the structures proposed for compounds **7** and **8**. The signal assignments in 1D spectra and the skeleton connectivity have been proven by 2D homo- and heteronuclear through bonds correlations. Information regarding the binding of the two cyclic moieties (piperidine and furanone) to the central naphthalimide skeleton was obtained from ^1^H-^15^N long range correlation NMR spectrum, as exemplified in Figure 1 for compound **7**. Thus, the piperidine nitrogen (73.6 ppm) has correlation signals with both naphthalimide H-5 proton (7.34 ppm) and methylene protons H-3″ (1.82 ppm), while naphthalimide nitrogen (223.6 ppm) has correlation signal with furanone proton H-2′ (6.60 ppm). The exact molecular weights were obtained by high resolution MS and the consistency of the molecular formulas was assessed by comparison with the experimental and simulated isotopic patterns. For both compounds **7** and **8**, the purity was demonstrated by ^1^H and ^13^C spectra, as presented in Supplementary Figures S1–S5 from Supplementary Information.

**Figure 2:**
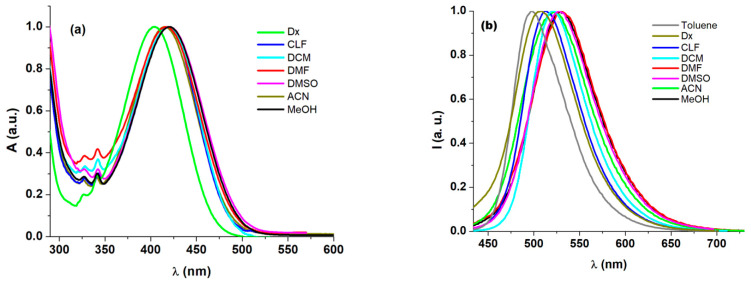
Normalized (**a**) UV-vis absorption and (**b**) fluorescence spectra (λ_ex_ = λ_a_) of **7** in different solvents.

The photophysical characteristics of the naphthalimide derivatives under study in terms of absorption maxima (ν_a_), emission maxima (ν_f_), Stokes shifts (Δν), and fluorescence quantum yields (Φ) were investigated in solvents with different polarities. The corresponding spectral data are presented in Figure 2 and Table 1. The electronic absorption spectra of naphthalimide derivatives show a broad absorption band in the visible range, located at 402 nm for **7**, in dioxane (Figure 2a). This absorption band can be assignable to a π-π* transition due to the naphthalimide moiety [10,47,48]. A shift to longer wavelength of visible absorption band was observed as the solvent polarity increased (Table 1). Thus, a red shift of 20 nm of the position of the absorption band of **7** was found for DMSO and this shift was of 16 nm in DCM relating to a non-polar solvent (dioxane). However, due to the electron-donating character of the piperidine unit, an intramolecular charge transfer from this group to the naphthalimide fragment can be generated [35,49]. The charge transfer character of this transition is proved by the red shift of the absorption band around 400 nm passing to a nonpolar solvent to a polar one. The high molar absorptivity (ε = 11,300 mol^−1^ Lcm^−1^, **8**) shows also that this electronic transition from the ground state to the excited state for naphthalimides possesses a π-π* character. As can be seen from the data comprised in Table 1, the solvent effect is more pronounced on the emission bands than on the absorption bands. In this case, the spectral shift owing to the high solvent polarity is much greater, increasing practically two fold relating to the absorption shift. The emission spectra of piperidine-naphthalimide derivatives obtained by excitation in the absorption band maximum exhibit an emission band in the range 495–540 nm depending on the solvent polarity (Figure 2b and Table 1). For example, the derivative **7** shows a yellow green emission at 495.5 nm in toluene, which is red shifted to 536 nm in DMF. These naphthalimides exhibit very strong emission in nonpolar solvents (dioxane, Φ = 0.821 (**7**); toluene, Φ = 0.453 (**8**); DCM, Φ = 0.530 (**8**)), while in polar solvents the quantum efficiency of emission decreases drastically (acetonitrile, Φ = 0.106 (**7**); DMSO, Φ = 0.003 (**8**)) (Table 1). The decrease of emission intensity in polar solvents can be assigned to a possible photoinduced electron transfer (PET) from the piperidine unit to the excited state of the naphthalimide fluorophore [35]. In this way, the emission of the naphthalimide derivatives 7, **8** in polar media is quenched. Recently, the decrease of the quantum yield in polar solvents was attributable to the presence of a twisted intramolecular charge transfer process in piperidine-naphthalimides, which leads to more stabilized excited states in polar solvents [49,50].

The energy yield of the fluorescence was estimated according to Equation (1). This parameter can also be utilized instead of quantum yield of fluorescence (Φ) [51,52].

(1) $$E_{F}=\Phi \frac{\lambda_{a}}{\lambda_{f}}$$

The calculated values of E_F_ are placed in the range 0.012–0.648 for **7** and 0.002–0.673 for **8** in solvents with different polarities (Table 2). It should be mentioned that the values of fluorescence quantum yield remained in the same range for both naphthalimide derivatives, **8** having the lowest quantum yield in polar solvents. Also, compound **7** presents higher energy yields of fluorescence.

**Figure 3:**
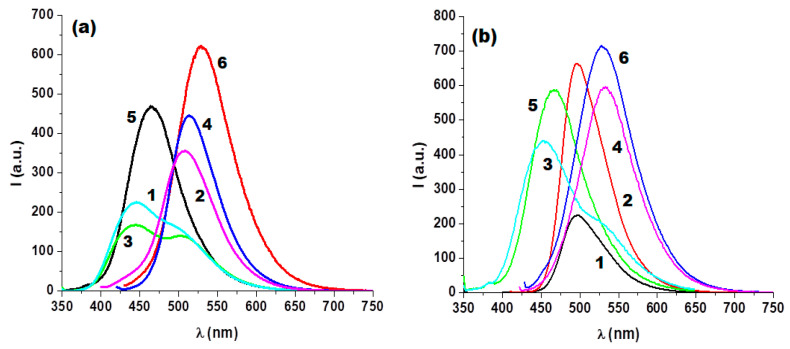
Fluorescence spectra of naphthalimide derivatives in different solvents as a function of excitation wavelength for (**a**) **7** (1—dioxane (Dx) (342 nm); 2—dioxane (Dx) (398 nm); 3—CHCl_3_ (343 nm); 4—CHCl_3_ (417 nm); 5—MeOH (342 nm); 6—MeOH (420 nm)) and (**b**) **8** (1—toluene (344 nm); 2—toluene (406 nm); 3—CH_3_CN (ACN) (341 nm); 4—CH_3_CN (ACN) (414 nm) (343 nm); 5—MeOH (342 nm); MeOH (420 nm)).

The effect of the excitation wavelength on the fluorescence spectra was analyzed. It is evident that the emission pattern is strongly dependent on the solvent nature, but the emission behavior of the two samples is different relating to the excitation wavelength. At the excitation with the wavelength corresponding to the visible absorption band, one emission band was found for the two naphthalimides according to Table 1. The emission pattern was changed at excitation with a wavelength of 340 or 327 nm. In this case, for **7**, besides the longer wavelength emission, a new emission band of lower intensity appeared in dioxane, chloroform, DCM, and DMSO in the spectral range of 430–480 nm. However, in methanol, acetonitrile, and DMF, a single emission band was observed in the range 440–465 nm (Figure 3a). For **8,** the longer wavelength emission band was also present in all solvents, excepting methanol, where at excitation with 342 nm, an emission band around 466 nm was found, and the emission band at longer wavelengths was not present in spectra. The intensity of the emission band around 465 nm is much higher in polar solvents, whereas in non-polar solvents this emission is very small (Figure 3b). The same behavior was observed for **7** and **8** at an excitation wavelength of 327 nm.

**Figure 4:**
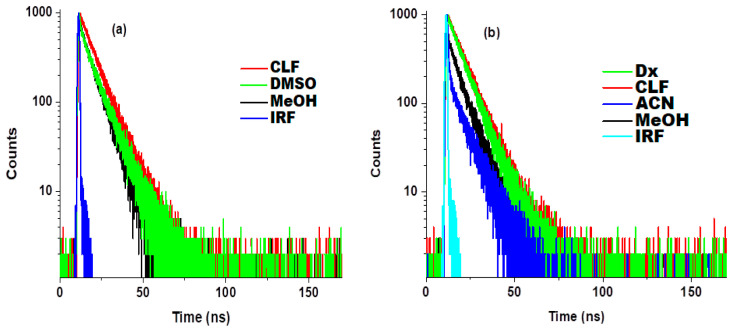
Fluorescence decay profiles of (**a**) **7** and (**b**) **8** in different solvents.

In order to find out more information about the excited state dynamics of the two piperidine-naphthalimides, time-resolved fluorescence experiments were made in solvents of different polarities. The values of the fluorescence lifetimes are listed in Table 3. Representative decay profiles of **7** in chloroform, methanol, DMSO; and of **8** in dioxane, chloroform, methanol, and acetonitrile are displayed in Figure 4. As can be seen from Table 3, the lifetime values were strongly affected by the solvent polarity. These naphthalimides exhibit a single exponential decay in solvents with low polarity (dioxane, toluene, chloroform, DCM) and in polar solvents the emission decay was fitted by a bi-exponential decay. The lifetime corresponding to the mono-exponential decay is around 8 ns for the two compounds in non-polar solvents (Table 3). This value is comparable with lifetimes of other naphthalimides, typical for fluorescent dyes [9].

In methanol, the measurements of the emission decay time indicated a longer time (7.68 ns) associated with a larger amplitude (93.51%) followed by a fast decay of 0.56 ns (amplitude 6.49%) for **7**, while for **8** the lifetime of the first component decay and the corresponding amplitude decrease significantly. The second decay component has a relatively longer lifetime and its amplitude becomes 85.96% in methanol. Moreover, the fluorescence decay profile of **8** is characterized by shorter lifetimes of the fast decay component and much higher amplitudes (Table 3). In polar solvents, the fitting of the experimental data shows a fast decay of 0.81 ns (amplitude 10.43%) for **7** in acetonitrile and of 0.55 ns (amplitude 7.93%) in DMSO. The long-lived components of **7** were around 9.00 ns with amplitude of approximately 90% in polar solvents. The emission decays of some naphthalimide derivatives fitted to biexponential curves in polar solvents were reported in literature [33,53]. The same pattern is valid for sample **8** in polar media, but the long-lived components have lower amplitudes (Table 3). The increase of the solvent polarity leads to the decrease of the fluorescence lifetimes and the same effect was found in the case of emission quantum yield (Table 1). The low amplitudes of short-lived components in polar media can be due to the enhanced charge transfer in polar solvents, and the short-lived components are not found out in solvents with low polarity. The increase of the nonradiative processes in the emission mechanism of piperidine-naphthalimides as the solvent polarity increases can be influenced by the intramolecular charge transfer mechanism.

The rate constants of radiative (k_r_) and nonradiative (k_nr_) decay can be estimated by the following relations:

(2) $$k_{r}=\frac{\Phi }{\tau }$$

(3) $$k_{nr}=\frac{1-\Phi }{\tau }$$

After having the values of τ_1_, τ_2_, a_1_, and a_2_, the average lifetime (τ_av_) was determined by the equation given below for a two-exponential decay:

(4) $$\tau_{av}=\frac{a_{1}\tau_{1}{}^{2}+a_{2}\tau_{2}{}^{2}}{a_{1}\tau_{1}+a_{2}\tau_{2}}$$

The values of the rate constants for the excited state decay by radiative and nonradiative pathways were summarized in Table 2. It could be observed that the values of k_r_ and k_nr_ practically are not dependent on the nature of the substituent of the piperidine unit, but they strongly depend on the solvent polarity. The effect of the solvent on the k_r_ values shows a nearly 50-fold decrease passing from dioxane to DMSO for **7** (Table 2). At the same time, the values of k_nr_ increased with the solvent changing from dioxane to DMSO, but the factor was smaller. The same pattern was found for derivative **8**. The lower values of the nonradiative rate constant reveals that these processes are important in polar protic solvents and the lifetimes are lower in these systems.

We correlated the microscopic solvent parameter E_T_(30) [54], which is utilized to describe the solvent effect on the spectral shifts, with quantum yield of naphthalimide derivatives. The evolution of the quantum yield of naphthalimides versus the solvent polarity parameter E_T_(30) is illustrated in Supplementary Figures S6 and S7. The quantum yield follows a decreasing trend when the solvent polarity increases. This specific behavior of the quantum yield suggests that the fluorescence mechanism is influenced by the solvent polarity, and a modification of the nonradiative decay rate can determine a significant decrease of the quantum yield values. The dependence of the radiative and nonradiative decay rates as a function of solvent polarity parameter E_T_(30) is given in Supplementary Figures S8–S10 for the two naphthalimides. As can be seen, an increase of the solvent polarity determines a progressive decrease of the k_r_ for the two naphthalimides, whereas an opposite trend is observed for k_nr_. This increasing trend of the k_nr_ corroborates that the photophysical response given by the quantum yield decreases in polar solvents. Thus, the interaction between solute presenting intramolecular charge transfer and solvent becomes more enhanced when the solvent polarity increases, which determines a decrease of the quantum yield. In this case, the increase of the nonradiatiave decay rate in the emission of these naphthalimides can be significantly influenced by intramolecular charge transfer (ICT) characteristics [55].

In order to study the solvatochromic behavior of naphthalimide derivatives, the Stokes shifts were correlated with solvent orientation polarizability, according to the expressions proposed by Lippert-Mataga and Bakhshiev as follows [56,57,58]:

(5) $$\Delta v=v_{a}-v_{f}=\frac{2{\left(\mu_{e}-\mu_{g}\right)}^{2}}{hca^{3}}\left[\frac{\varepsilon -1}{2\varepsilon +1}-\frac{n^{2}-1}{2n^{2}+1}\right]+const$$

(6) $$\Delta v=v_{a}-v_{f}=\frac{2{\left(\mu_{e}-\mu_{g}\right)}^{2}}{hca^{3}}\left[\frac{\varepsilon -1}{\varepsilon +2}-\frac{n^{2}-1}{\left(n^{2}+2\right)\cdot (2n^{2}+1)}\cdot \frac{1}{n^{2}+2}\right]+const$$

where ν_a_ and ν_f_ denote the wavenumbers of absorption and emission maxima, ε is the dielectric constant, *n* is the refractive index of the solvent, *h* is the Planck constant, c is the light velocity in vacuum, a represents the Onsager cavity radius around chromophore, and μ_g_ and μ_e_ are the ground and excited-state dipole moments. The correlation between Stokes shift (Δν) and the orientation polarizability functions $\frac{\varepsilon -1}{2\varepsilon +1}-\frac{n^{2}-1}{2n^{2}+1}$ and $\left[\frac{\varepsilon -1}{\varepsilon +2}-\frac{n^{2}-1}{n^{2}+2}\right]\cdot \frac{2n^{2}+1}{n^{2}+2}$ was found to be rather poor (Supplementary Figures S11 and S12). The deviation from the linearity can be due to the specific interactions between solvent and solute such as hydrogen bondings or intramolecular charge transfer, which can become important interactions in solvation. The hydrogen-bond donor solvents can give hydrogen bonds to the carbonylic oxygens of the naphthalimide.

For a better approach of the solvatochromism of naphthalimides, the microscopic solvent polarity, E_T_(30), which takes into account the polarity and acidity of solvent [54], was applied. The correlation between E_T_(30) and Δν does not obey a linear dependence, practically the solvents are placed in two distinct groups for the two samples (Supplementary Figures S13 and S14), suggesting two excited states in the photophysics of these derivatives [33]. The failure of the Lippert-Mataga correlation and of the E_T_(30) scale to describe the solvent dependence indicates that the hydrogen bonds can be responsible for the solvent shifts of ν_a_, ν_f_, or Δν of **7** and **8**.

In order to quantify the solvent effect on absorption, emission, and Stokes shifts energies, multiple linear regression (MLR) analysis employing different solvatochromic parameters given by Catalan scale [59,60], Kamlet–Taft scale [61,62,63] and [f(n), f(ε), β, α] scale [64,65] were used according to the equations:

(7) $$y=y_{0}+a_{SA}SA+b_{SB}SB+c_{SP}SP+d_{SdP}SdP$$

(8) $$y=y_{0}+aa+b\beta +c\pi *$$

(9) $$y=y_{0}+c_{1}f\left(n\right)+c_{2}f\left(\varepsilon \right)+c_{3}\beta +c_{4}\alpha$$

where *y* denotes the spectral property under discussion; *y*_0_ is the corresponding spectral property in gaseous phase; *SA*, *SB*, *SP,* and *SdP* represent acidity, basicity, polarizability and dipolarity of solvent; *a_SA_*, *b_SB_*, *c_SP_*, and *d_SdP_* are the corresponding regression coefficients; π* is the solvent dipolarity/polarizability; *α* is the hydrogen bond donating ability; *β* is the hydrogen bond accepting ability of the solvent; and the coefficients *a*, *b*, *c* indicate the contribution of the solvent parameters to the spectral shift. In relation 9, $f\left(\varepsilon \right)=\frac{\varepsilon -1}{\varepsilon -2}$, polarity function and $f\left(n\right)=\frac{n^{2}-1}{n^{2}+2}$ electronic polarizability function are included. The values of solvatochromic parameters [58,60,63,66] were collected in Table 4.

The results illustrated in Table 5, Table 6 and Table 7 are obtained following the multiple regression analysis of absorption maxima (ν_a_), emission maxima (ν_f_), and Stokes shifts (Δν) for derivatives **7** and **8**, according to Equations (7)–(9). The negative or positive values of different coefficients in the multilinear correlation analysis reveal the positive or negative solvatochromism of the compounds under study. From the multiple linear regression analysis, different interactions, which can be analyzed with Catalan, Kamlet–Taft and [f(n), f(ε), β, α] parameters, contribute differently to the absorption and emission band shifts. It is evident from Table 5, Table 6 and Table 7 that the regression coefficients (*c_SP_*, *d_SdP_*), (*c_1_*, *c_2_*) and *c* present higher values than (*a_SA_*, *b_SB_*), (*c_3_*, *c_4_*) and (*a*, *b*), which show the greater importance of the solvent polarizability and solvent dipolarity to the solvatochromism. Although the non-specific interactions in naphthalimide derivatives with solvents are the main factors affecting the spectral properties, the contributions of the medium acidity and medium basicity cannot be ignored. The negative algebraic sign of the regression coefficients of polarity and polarizability (excepting ν_f_) indicates the red shifts in the emission and absorption spectra for the three solvent scales, suggesting a greater dipole moment in the electronically excited state than the ground state. Moreover, the higher values of c_1_ and c_SP_ indicate that the dipolarity is of major importance in the solute–solvent interactions for **7**, while for **8** the polarizability is more definitive. The introduction of the benzyl group to piperidine moiety leads to the increase of the coefficient corresponding to polarizability in absolute value and the coefficient due to dipolarity varies little for **7** and **8** in the Catalan and [f(n), f(ε), β, α] scales.

**Figure 5:**
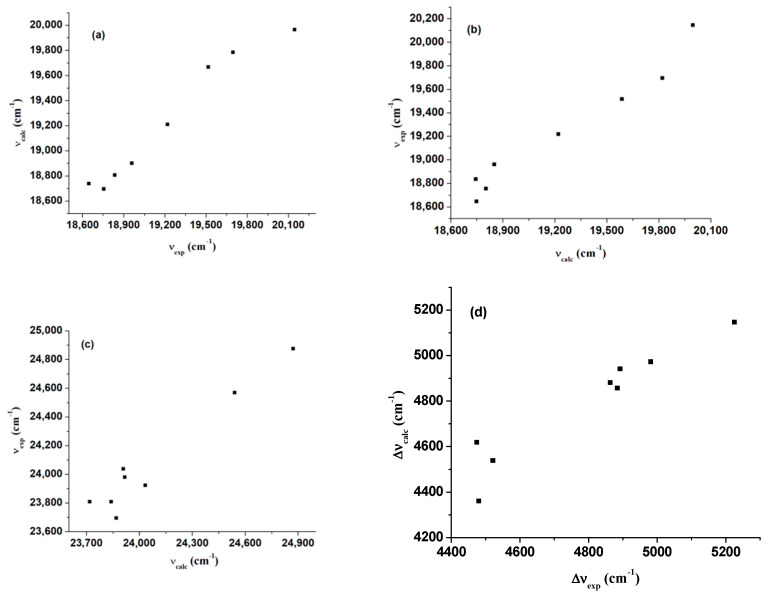
Linear relationship between the experimental maxima and (**a**) predicted fluorescence maxima using multilinear regression with [f(n), f(ε), β, α] scale for **7**, (**b**) fluorescence frequencies estimated using Catalan parameters for **8**, (**c**) calculated ν_a_ of **7** using Kamlet–Taft scale, and (**d**) predicted Δν using [f(n), f(ε), β, α] scale for **7**.

It is found that the regressions performed with the three solvent scales afford practically similar values for y_0_, indicating that the photophysical characteristics given by these models for **7** and **8** are rather close. Within [f(n), f(ε), β, α] approach, the best linear regressions were obtained for ν_a_, ν_f_, and Δν for **7** and **8** (R^2^ > 0.94) (Table 7). Also, the Catalan scale gives a good fit of absorption and emission maxima of the two naphthalimides (R^2^ > 0.93) (Table 5). The Kamlet–Taft solvent scale gives modest correlations for Δν (**7**), ν_f_ (**8**) and Δν (**8**) (Table 6). The weaker performance of Kamlet–Taft regression can be due to the fact that the solvent dipolarity and solvent polarizability are contained in π* parameter, whereas in Catalan model these indicators are separated [67]. The values of the regression parameters corresponding to the Catalan, Kamlet–Taft, and [f(n), f(ε), β, α] scales indicate the sensitivity of spectral parameters ν_a_, ν_f_, or Δν of **7** and **8** to the solvent polarity, acidity, and basicity. An accurate analysis is proved by a good correlation between the theoretical values of these spectral parameters obtained by the estimated regression coefficients and the experimental values (Figure 5 and Supplementary Figures S15–S18).

**Figure 6:**
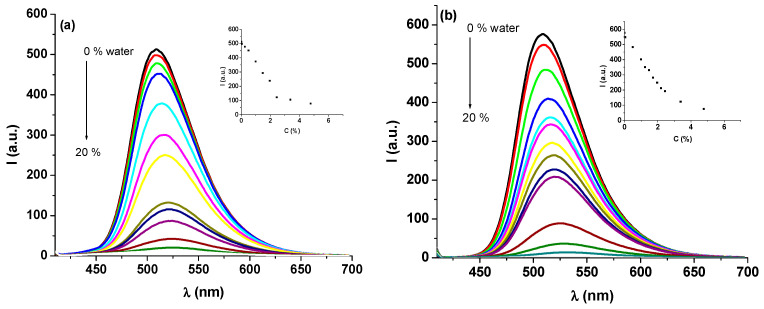
Fluorescence spectra in dioxane upon gradual addition of different amounts of water (λ_ex_ = 402 nm) for (**a**) **7** and (**b**) **8**. Insets present the linear dependence of emission intensity versus water level.

The nonradiative TICT (twisted intramolecular charge transfer) process in piperidine-naphthalimides is very sensitive to the presence of water in solution because the emission intensity of **7** and **8** is strongly dependent on its surroundings. The addition of water in dioxane solutions of **7** and **8** determines the decrease of fluorescence intensity of the two naphthalimides with a red shift of the emission maxima. The emission spectra of **7** and **8** in dioxane at different water levels are given in Figure 6. The effect of fluorescence quenching was observed even for low levels of water. The fluorescence quenching efficiency was determined by the expression (I_o_ − I)/I_o_, where I_o_ and I represent the emission intensity before and after the addition of water as quencher. The most dramatic changes of the emission intensity of the two naphthalimide were registered at low water concentrations of less than 2%, which means that a quenching efficiency of 66.6% and 76.2% was obtained for **7** and **8**, respectively, at a water level of 2.43%. Moreover, at higher water contents (>10%), the emission of **7** and **8** practically was quenched (Figure 6). Also, the fluorescence quantum yield decreases as the water level increases in dioxane solutions. As the water content in the dioxane/water system increases to 2.44%, the quantum yield becomes 0.418 for **7** and 0.485 for **8**. The further increase of the water level in solution leads to a considerable decrease of the quantum yield, reaching 0.037 for **7** and 0.034 for **8**, respectively, at a water content of 20%. The solvent (dioxane) is non-polar but, by the presence of water in solution, the mixture becomes more polar and the quantum yield decreases significantly as mentioned previously [33]. In polar solvents (DMF, DMSO), the fluorescence quantum yield for both naphthalimides practically is not influenced by the increase of the water level, as compared to the values corresponding to neat solvent (Table 1).

**Figure 7:**
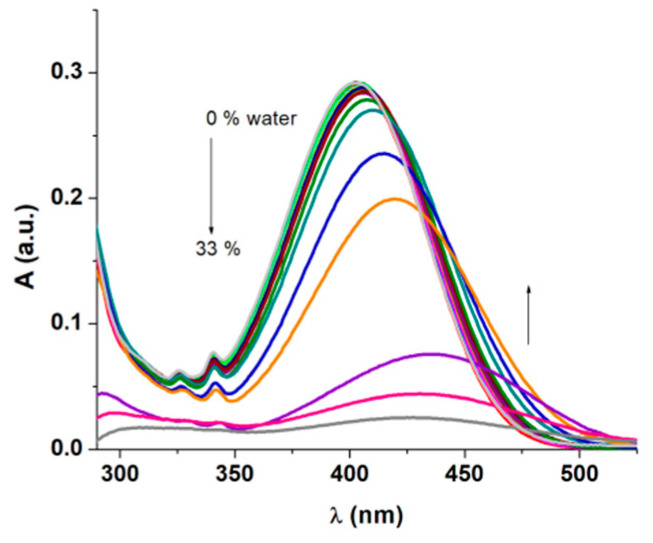
Changes in the electronic absorption spectra of **8** in dioxane solution as a function of water level.

The electronic absorption spectra of green-emitting derivatives **7** and **8** in dioxane are displayed in Figure 7 and Supplementary Figure S19, respectively. The increase of water content in solution causes the decrease of the absorbance around 400 nm by 30%, and a bathochromic shift to 425 nm of this absorption band was observed (Supplementary Figure S19). The same features were observed for **8**, but in this case, the intensity of the absorption band around 400 nm exhibits a decrease of almost 90% accompanied by the same red shift (Figure 7). In both cases, a broadening of this absorption band was noticed due to the H bonding interactions between imidic carbonyl groups and solvent [32]. It should be pointed out that the fluorescence intensity of **7** and **8** in dioxane/water system varies almost linearly as the water level increases in the first stages of water adding up to 3% (Figure 6 inset), suggesting that the **7** and **8** derivatives are sensitive to the polarity changes of the microenvironment.

**Figure 8:**
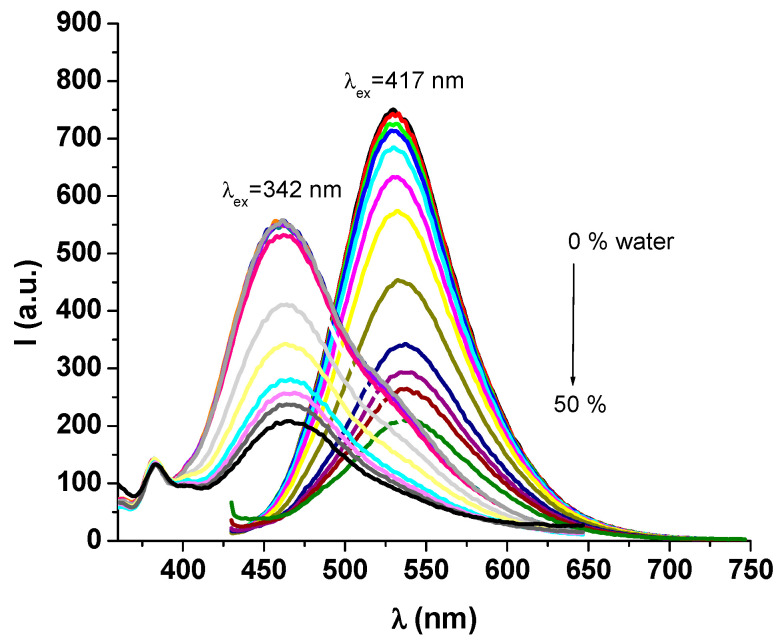
Fluorescence spectra of **8** in DMF adding different water levels (λ_ex_ = 420 nm, 342 nm).

**Figure 9:**
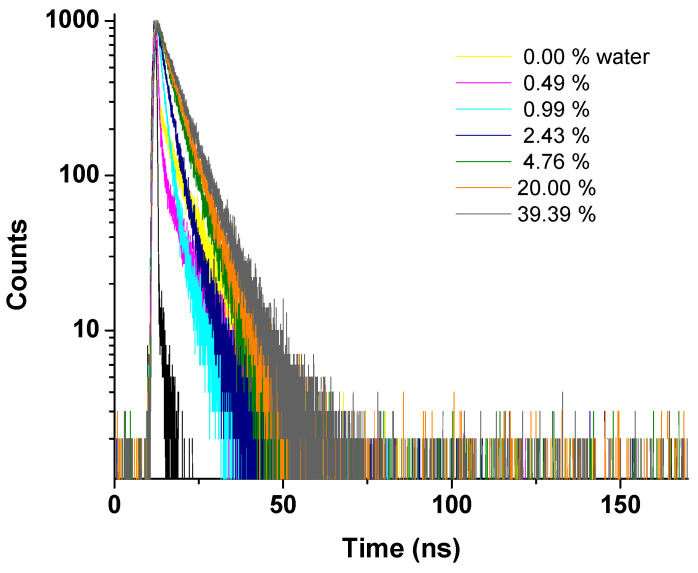
Fluorescence decay curves of **8** in dioxane with different amounts of water.

To explore the ability of naphthalimide derivatives to detect water in organic solvents, other solvents such as DMF and DMSO were utilized. Also, the emission intensity of **7** and **8** decreases progressively as the water level in solution increases, exciting with 340 or 417 nm (Figure 8, Supplementary Figures S20 and S21). It should be noticed that these derivatives show lower water sensitivity, because the estimated quenching efficiency values were 24.8% (DMF), and 13.1% (DMSO) for **7;** and 24.2% (DMF) and 15.2% (DMSO) for **8**, respectively, for a water content of 9.1%. The quenching efficiency reaches 60% (DMF) and 50% (DMSO) for a water content of 40%. The addition of water to DMF and DMSO solutions of **7** and **8** induces also the decrease of absorbance at about 417 nm and the red shift of the absorption band, similarly to dioxane solutions. For example, a red shift from 417 nm to 430 nm was observed in DMF for **7** and from 429 nm to 433 nm in DMSO for **8**, in the electronic absorption spectra. The bathochromic shift of the emission maxima of the two naphthalimides by increasing the water content is in line with previously reported data [2,20]. The addition of the water amounts to over 60% in DMF solution of **8,** which leads to the increase of the emission intensity up to three times and the emission band shifts to longer wavelengths around 548 nm, under 417 nm excitation wavelength. Another emission channel was found out when the excitation was performed at 340 nm. In this case, the emission band located at 463 nm also decreased in intensity when water was added to the system; this band disappeared practically for high water levels and a new weak emission band appeared around 545 nm (Figure 9). For high water levels in mixture, the absorption band at 417 nm disappeared. In nonpolar solvents, a single emission channel was present, regardless of the exciting wavelengths. This specific solvatochromic behavior of the naphthalimides was evinced in 3D fluorescence spectra obtained in solvents with different polarities (Supplementary Figure S22).

The fluorescence decay profiles of **7** and **8** in dioxane/water mixtures are displayed in Figure 9 and Supplementary Figure S23. For low water fractions (<2%), the emission decay can be described by a monoexponential model. For higher water levels, the fluorescence decay profiles were fitted to a biexponential model (Table 8). Analyzing the fluorescence lifetimes of **7** and **8** derivatives, it can be noticed that the value of the short-lived emission component decreases as the water level increases, and their corresponding amplitudes increase, while the long-lived emission presents a slight increase, but their amplitudes decrease as the water fraction increases (Table 8).

When the water level of the DMF/water solution increases from 0 to 30%, the short-lived emission component in the fluorescence decay curve increases (Supplementary Figures S24 and S25), whereas the long-lived component decreases, but their amplitudes practically remain unchanged for **7**. The same pattern was kept for derivative **8**, but in this case the amplitude of short-lived component significantly decreases (around 50%), whereas the long-lived component increases at about 20% (Table 9).

The piperidine-naphthalimides **7** and **8** were investigated to detect the dichloromethane from chloroform. However, in this case the quenching efficiency was found to be around 30% for the two derivatives (Supplementary Figures S26 and S27), and these compounds could not act as a fluorescence sensor for dichloromethane determination.

The fluorescence quenching data were discussed using Stern–Volmer equation [68,69,70]:

(10) $$\frac{I_{0}}{I}=1+K_{SV}\left[Q\right]$$

where *I*_o_ and *I* denote the emission intensity in the absence and presence of the quencher, *K_SV_* is the Stern–Volmer quenching constant, and [*Q*] is the quencher concentration.

**Figure 10:**
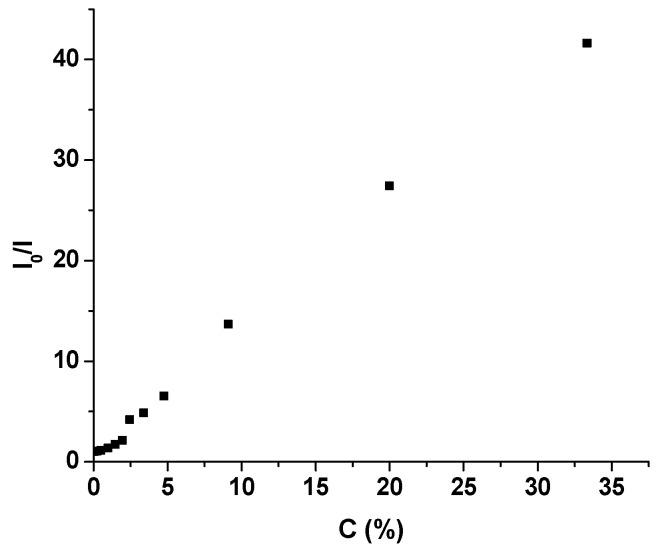
Stern–Volmer curves for fluorescence quenching of **7** in dioxane adding different amounts of water.

The Stern–Volmer (S-V) plot upon water addition to dioxane solution of **7** is linear over the whole concentration range (Figure 10), whereas the S-V plots for DMF/water and DMSO/water are nonlinear, exhibiting an upward deflection with the respect to the ordinate axis as shown in Supplementary Figures S28 and S29. The S-V constant, K_SV_, was determined from the slope of the linear plot of I_o_/I versus [quencher]. The S-V plots upon the addition of water in dioxane, DMF, and DMSO solutions of **8,** exhibit also an upward curvature at high water levels, with a dominant linear relationship at lower water content (Supplementary Figures S30–S32).

The fluorescence quenching upon the addition of water can occur by a static or dynamic process or by a combination of these two processes. The linearity of the S-V plots obtained from state-steady determinations indicates that the quenching process can take place by a single mechanism, either static or dynamic [68,70,71]. In the present case, the S-V curves were found to be nonlinear, presenting an upward curvature with respect to the Oy axis, which suggests that the emission of these naphthalimides was quenched by both dynamic and static processes. However, the quenching occurring for **7** in dioxane/water mixture due to the linearity of the data on the whole range of water content can be presumed to have preponderantly a static nature, but the decrease of fluorescence lifetime during water addition imply the existence of a dynamic mechanism for **7** (Figure 10). The decrease of the emission lifetimes with increasing water level can be due to an enhanced ICT in these derivatives [72].

It is observed that in the absence and the presence of water as quenching agent, the absorption and emission spectra show observable differences in their shape and position of band maxima, which suggest that the quenching process reveals more complicated features. The values of K_SV_ are higher for dioxane/water system for both derivatives (Table 10), showing an efficient quenching in this mixture as compared to DMF/water and DMSO/water mixtures. The fluorescence spectra of **7** and **8** in DMF solution were obtained as a function of temperature in the 25–55 °C range. The emission maxima are not shifted with temperature, so the emission state is not affected by heating. Generally, a small decrease in intensity was observed in this temperature range, being below 10% in DMF and 20%, respectively, in DMSO for **8** (Supplementary Figure S33).

The detection limit (LOD) was estimated by the relation LOD = 3σ/k, where σ is the standard deviation (SD) of the fluorescence intensity of the sample for 10 blank measurements, and k is the slope of the linear fit of fluorescence intensity versus water content. Supplementary Figure S34 shows the plot of the fluorescence intensity as a function of the water content for 8 over a response range of 0–5%. The detection limit of piperidine-naphthalimide 8 to water in dioxane was estimated to be 0.151% using the standard deviation of blank sample and the slope of the linear regression fit. It is noteworthy that the detection limit is close to the values previously reported [35,73].

**Figure 11:**
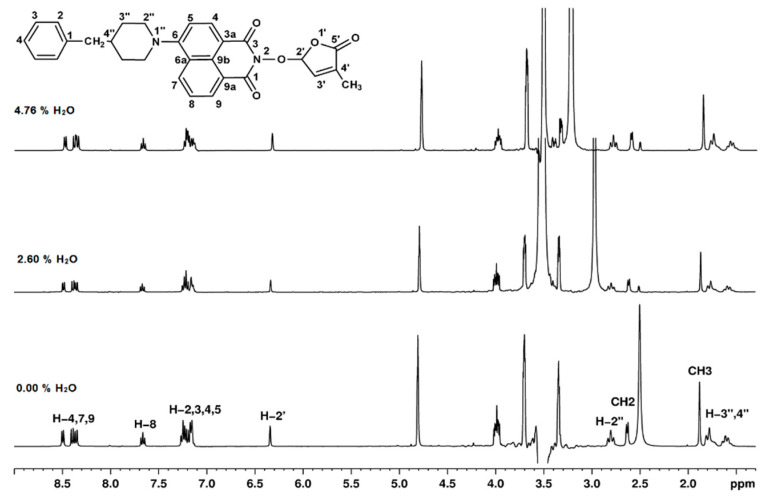
^1^H-NMR spectra recorded with dioxane suppression for compound **8** before and after the addition of 2.60% and 4.76% water content.

The stability of piperidine-naphthalimides in dioxane/water mixture was verified through NMR titration for compound **8**. For these experiments, a small amount of compound **8** was dissolved in non-deuterated dioxane, using a DMSO-d6 capillary as lock solvent for NMR. Proton spectra were recorded with dioxane suppression after the addition of 0.33% water, until a total water content of 4.76% was reached. The initial point (before the water addition), middle, and final points for these titration experiments are presented in Figure 11. As it can be observed, there are no significant changes in the proton NMR signals, indicating that compound **8** is stable in the presence of small amounts of water. The above-mentioned results suggested that naphthalimide derivatives **7** and **8** could operate as fluorescence sensors for water trace determination.

## 3. Materials and Methods

### 3.1. Materials

The intermediate compounds, 6-amino-benzo[*de*]isochromene-1,3-diones **2** and **3**, and 2-hydroxy-6-amino-benzo[*de*]isoquinoline-1,3-diones **4** and **5**, were obtained according to the reported procedures [47]. The common intermediate, 5-bromo-3-methyl-5*H*-furan-2-one **6**, was prepared in good yield by the bromination of 3-methyl-5*H*-furan-2-one with *N*-bromosuccinimide in carbon tetrachloride by a previously described method [74]. All others reagents were commercial products (Sigma-Aldrich) and were used as received, without further purification. All solvents (Sigma-Aldrich and Merck) were pure or of spectroscopic grade.

### 3.2. General Procedure for the Synthesis of 2-(4-Methyl-5-oxo-2,5-dihydrofuran-2-yloxy)-6-(4-methylpiperidin-1-yl)-benzo[de]isoquinoline-1,3-dione (***7***) and 2-(4-Methyl-5-oxo-2,5-dihydrofuran-2-yloxy)-6-(4-benzyl-piperidin-1-yl)-benzo[de]isoquinoline-1,3-dione (***8***)

To a solution of 6-amino-2-hydroxy-benzo[*de*]isoquinoline-1,3-dione **4** or **5** (5 mmol) and anhydrous K_2_CO_3_ (1.38 g, 10 mmol) in 15 mL of DMF, crude 5-bromo-3-methyl-5*H*-furan-2-one **2** (1.25 g, 7 mmol) was added under stirring at room temperature. The mixture was stirred at room temperature for 24 h. The reaction mixture was poured in 100 mL water and extracted with 3 × 150 mL CHCl_3_, combined with extracts washed with an equal volume of water and dried on anhydrous Na_2_SO_4_. The solvent was partly removed under reduced pressure and the solid formed was filtered and recrystallized to obtain compounds **7** or **8**.

### 3.3. Characterization Data

#### 3.3.1. 2-(4-Methyl-5-oxo-2,5-dihydrofuran-2-yloxy)-6-(4-methylpiperidin-1-yl)-benzo[*de*]isoquinoline-1,3-dione (**7**)

Yield: 62% (1.26 g), m.p.: 198–200 °C (CHCl_3_), Orange crystals.

Anal.: Calcd. for C_23_H_22_N_2_O_5_ (406.44): C, 67.97; H, 5.46; N, 6.89%. Found: C, 68.10; H, 5.54; N, 5.93%.

IR (KBr, ν_max_, cm^−1^): 3432, 2916, 1784, 1715, 1676, 1584, 1457, 1363, 1229, 1193, 1088.

^1^H NMR (600 MHz, DMSO-d6): δ (ppm) = 1.04 (d, *J* = 6.4 Hz, 3H, CH_3_-4″), 1.52 (dd, *J* = 22.6, 11.2 Hz, 2H, CH_2_-3″A), 1.63–1.65 (m, 1H, CH-4″), 1.82 (d, *J* = 11.9 Hz, 2H, CH_2_-3″B), 1.92 (bs, 3H, CH_3_-4′), 2.93 (t, *J* = 11.9 Hz, 2H, CH_2_-2″A), 3.56 (d, *J* = 11.9 Hz, 2H, CH_2_-2″B), 6.60 (bs, 1H, H-2′), 7.34 (d, *J* = 8.2 Hz, 1H, H-5), 7.47 (bs, 1H, H-3′), 7.83 (t, *J* = 7.8 Hz, 1H, H-8), 8.40 (d, *J* = 8.1 Hz, 1H, H-4), 8.44 (d, *J* = 8.4 Hz, 1H, H-7), 8.50 (d, *J* = 7.1 Hz, 1H, H-9).

^13^C NMR (150.9 MHz, DMSO-d6): δ (ppm) = 10.2 (CH_3_-4′), 21.7 (CH_3_-4″), 30.2 (CH-4″), 33.9 (CH_2_-3″), 53.2 and 53.3 (CH_2_-2″ and CH_2_-6″), 104.0 (CH-2′), 114.6 (C-3a), 115.1 (CH-5), 122.7 (C-9a), 125.5 (C-6a), 125.9 (CH-8), 128.7 (C-9b), 131.1 (CH-9), 131.4 (CH-7), 132.8 (CH-4), 134.2 (C-4′), 141.9 (CH-3′), 157.2 (C-6), 159.9 (CO-3), 160.4 (CO-1), 171.0 (CO-5′).

^15^N NMR (60.8 MHz, DMSO-d6): δ(ppm) = 73.6 (N-1″), 223.6 (N-2).

HRMS-ESI (*m*/*z*): [M + Na]^+^ for C_23_H_22_N_2_NaO_5_, calcd. 429.1421, found 429.1425.

#### 3.3.2. 2-(4-Methyl-5-oxo-2,5-dihydro-furan-2-yloxy)-6-(4-benzyl-piperidin-1-yl)-benzo[*de*]isoquinoline-1,3-dione (**8**)

Yield: 58% (1.40 g), m.p.: 182–184 °C (CHCl_3_), Orange crystals.

Anal.: Calcd. for C_29_H_26_N_2_O_5_ (482.54): C, 72.19; H, 5.43; N, 5.81%. Found: C, 72.06; H, 5.33; N, 5.93%.

IR (KBr, ν_max_, cm^−1^): 2911, 2794, 1784, 1718, 1684, 1584, 1452, 1364, 1235, 1177, 1026.

^1^H NMR (600 MHz, DMSO-d6): δ (ppm) = 1.58 (dd, *J* = 22.6, 11.2 Hz, 1H, CH_2_-3″A), 1.77 (d, *J* = 11.3 Hz, 3H, CH_2_-3″B and CH-4″), 1.91 (bs, 3H, CH_3_-4′), 2.65 (d, *J* = 6.5 Hz, 2H, CH_2_-4″), 2.87 (t, *J* = 12.1 Hz, 2H, CH_2_-2″A), 3.56 (d, *J* = 11.6 Hz, 2H, CH_2_-2″B), 6.60 (bs, 1H, H-2′), 7.21 (t, *J* = 7.2 Hz, 1H, H-4Ph), 7.24 (d, *J* = 7.3 Hz, 2H, H-2Ph), 7.31 (d, *J* = 7.2 Hz, 1H, H-5), 7.32 (t, *J* = 8.0 Hz, 2H, H-3Ph), 7.46 (bs, 1H, H-3′), 7.82 (t, *J* = 7.9 Hz, 1H, H-8), 8.39 (d, *J* = 8.2 Hz, 1H, H-4), 8.42 (d, *J* = 8.3 Hz, 1H, H-7), 8.49 (d, *J* = 7.2 Hz, 1H, H-9).

^13^C NMR (150.9 MHz, DMSO-d6): δ (ppm) = 10.3 (CH_3_-4′), 31.8 (CH_2_-3″), 37.3 (CH-4″), 42.3 (CH_2_-4″), 53.2 and 53.3 (CH_2_-2″ and CH_2_-6″), 104.0 (CH-2′), 114.7 (C-3a), 115.1 (CH-5), 122.7 (C-9a), 125.5 (C-6a), 125.9 (CH-4Ph), 126.0 (CH-8), 128.2 (CH-3Ph), 128.7 (C-9b), 129.1 (CH-2Ph), 131.2 (CH-9), 131.4 (CH-7), 132.8 (CH-4), 134.2 (C-4′), 140.2 (C-1Ph), 141.9 (CH-3′), 157.1 (C-6), 160.0 (CO-3), 160.5 (CO-1), 171.1 (CO-5′).

^15^N NMR (60.8 MHz, DMSO-d6): δ(ppm) = 74.2 (N-1″), 223.4 (N-2).

HRMS-ESI (*m*/*z*): [M + Na]^+^ for C_29_H_26_N_2_NaO_5_, calcd. 505.1734, found 505.1738.

### 3.4. Methods

Melting points were determined on a Boetius apparatus and are uncorrected. The IR absorption spectra were recorded on a Nicolet Impact 410 spectrometer (Thermo Electron Scientific Instruments, Madison WI, USA), in KBr pellets. The NMR analyses were performed on Bruker Avance Neo spectrometers (Bruker Biospin, Ettlingen, Germany), operating at 600.1, 150.9, and 60.8 MHz for ^1^H, ^13^C, and ^15^N, respectively. Chemical shifts are reported in δ units (ppm) and were referenced to the residual solvent signals (DMSO-d6 ^1^H at 2.51 ppm and ^13^C at 39.4 ppm). The ^15^N chemical shifts are referenced to liquid ammonia (0.0 ppm) using nitromethane (380.2 ppm) as external standard. The 1D and 2D spectra were recorded with a 5 mm multinuclear inverse detection z-gradient probe. Unambiguous 1D NMR signal assignments were made based on 2D NMR homo- and heteronuclear correlations. H-H COSY, H-C HSQC, and H-C HMBC experiments were recorded using standard pulse sequences in the version with z-gradients, as delivered by Bruker with TopSpin 4.0.8 spectrometer control and processing software. The ^15^N chemical shifts were obtained as projections from the 2D indirectly detected H-N HMBC spectra, employing a standard pulse sequence in the version with z-gradients as delivered by TopSpin 4.0.8 (Bruker Biospin, Ettlingen, Germany). Exact molecular weights were obtained from high resolution MS spectra recorded on a Bruker Maxis II QTOF spectrometer with electrospray ionization (ESI) in the positive mode.

Ultraviolet-visible absorption spectra were taken on a SPECORD 210Plus spectrophotometer (Analytik Jena, Jena, Germany). The fluorescence spectra were obtained using a Perkin Elmer LS55 (PerkinElmer, Inc., Waltham, Ma, USA) spectrometer or an Edinburgh FS5 spectrofluorometer (Edinburgh Instruments, Livingston, UK). The time resolved fluorescence measurements were carried out on an Edinburgh FLS980 spectrofluorometer. The excitation source was a nanosecond diode laser at 405 nm. The time resolved transients were fitted by using single or double exponential functions, $I\left(t\right)=a_{1}e^{-\frac{t}{\tau_{1}}}+a_{2}e^{-\frac{t}{\tau_{2}}}$, where *I*(*t*) represents the emission intensity at time *t*, and *a_i_* and *τ_i_* are the preexponential factor and the component t of the decay time, respectively. The best fitted parameters were estimated by the minimization of the reduced χ^2^ value and of the residual distribution of the experimental data. Fittings having chi-squared values around 1 and symmetrical distributions of the residual were accepted. The emission quantum efficiency (Φ) was measured using a FLS980 integrating sphere for dilute solutions (A < 0.1) at the excitation wavelengths corresponding to visible absorption band. All spectral measurements were performed at room temperature, and 10 mm path length quartz cells were utilized for spectroscopic determinations. The thin films for solid-state photophysical measurements were prepared onto quartz plates from dilute dichloromethane solution by dipping method. The thin films were kept dry and left to evaporate the solvent afterwards.

## 4. Conclusions

Novel fluorescent strigolactone mimics containing piperidine-substituted 1,8-naphthalimides linked by an ether bond to a bioactive 3-methyl-furan-2-one fragment were synthesized and their spectral and emission properties were discussed. These compounds are highly fluorescent in nonpolar solvents (dioxane, dichloromethane) and the emission intensity decreased drastically in polar solvents (acetonitrile, DMF, DMSO). When water was added in the dioxane solutions, the emission intensity of solution was rapidly quenched. The quenching effect on emission intensity was also observed in polar solvents, but for higher water levels. Catalan and [f(n), f(ε), β, α] multiparametric solvent scales revealed that the solvent dipolarity and polarizability have a significant impact on the solvatochromic shifts of the absorption and emission maxima. The obtained results suggest that these naphthalimide-based derivatives can act as a potential sensor detecting low amounts of water. The emission efficiency is strongly dependent on the solvent polarity. A fast nonradiative deactivation of the emission was observed in polar solvents due to enhancing intramolecular charge transfer process. The fluorescence quenching process was illustrated by nonlinear S-V plots and reduced excited lifetimes in the water presence. Fluorescence quenching can be identified as a combined static and dynamic process.

## Figures and Tables

**Table 1:** Optical properties of piperidine-naphthalimide derivatives in different solvents.

Solvent	E_T_(30)	7				8			
		ν_a_ (cm^−1^)	ν_f_ (cm^−1^)	Δν (cm^−1^)	Φ	ν_a_ (cm^−1^)	ν_f_ (cm^−1^)	Δν (cm^−1^)	Φ
Toluene	33.9	24,570	20,096	4474	0.546	24,691	20,145	4546	0.453
Dioxane	36.0	24,785	19,650	5225	0.821	24,875	19,696	5179	0.875
Chloroform	39.1	23,980	19,500	4480	0.686	23,980	19,516	4464	0.726
DCM	40.7	23,696	19,175	4521	0.451	23,752	19,219	4533	0.529
DMF	43.2	23,923	18,942	4981	0.214	23,923	18,646	5277	0.064
DMSO	45.1	23,809	18,925	4884	0.015	23,809	18,835	4974	0.003
Acetonitrile	45.6	24,038	19,175	4863	0.106	24,154	18,754	5400	0.017
Methanol	55.4	23,809	18,917	4892	0.082	23,866	18,960	4906	0.081

**Table 2:** Photophysical data of piperidine-naphthalimides in different solvents.

Solvent	7			8		
	k_r_(×10^−7^) (s^−1^)	k_nr_(×10^−7^) (s^−1^)	E_F_	k_r_(×10^−7^) (s^−1^)	k_nr_(×10^−7^) (s^−1^)	E_F_
Toluene	6.86	5.70	0.447	5.84	7.06	0.369
Dioxane	10.15	2.21	0.648	10.87	1.55	0.673
DCM	5.39	6.57	0.366	6.28	5.58	0.426
DMF	2.46	9.02	0.171	0.75	12.39	0.049
DMSO	0.17	11.29	0.012	0.38	11.92	0.002
Acetonitrile	1.17	9.85	0.085	0.20	10.31	0.013
Methanol	1.07	12.01	0.065	1.11	12.66	0.065

**Table 3:** Fluorescence lifetimes of piperidine-naphthalimides in different solvents.

Solvent	7				8			
	τ_1_ (ns)/a_1_(%)	τ_2_(ns)/a_2_(%)	χ^2^	τ_av_ (ns)	τ_1_(ns)/a_1_(%)	τ_2_(ns)/a_2_(%)	χ^2^	τ_av_ (ns)
Toluene	7.96 (100)		0.99	7.96	7.75 (100)		1.00	7.75
Dioxane	8.09 (100)		1.00	8.09	8.05 (100)		1.00	8.05
Chloroform	8.69 (100)		1.00	8.69	8.49 (100)		1.00	8.49
DCM	8.36 (100)		1.00	8.36	8.43 (100)		1.00	8.43
DMF	0.55 (7.93)	8.76 (92.07)	0.99	8.71	0.21 (41.46)	7.75 (58.54)	1.00	7.61
DMSO	0.85 (10.00)	8.80 (90.00)	1.00	8.72	0.36 (21.68)	8.23 (78.32)	1.00	8.13
Acetonitrile	0.81 (10.43)	9.17 (89.57)	1.00	9.08	0.44 (40.32)	8.36 (59.68)	1.00	8.08
Methanol	7.68 (93.51)	0.56 (6.49)	1.00	7.65	0.26 (14.04)	7.29 (85.96)	1.00	7.26

**Table 4:** Solvents and solvent properties.

No.	Solvent	ε	n	π*	β	α	SP	SdP	SB	SA	E_T_(30)
1.	Toluene	2.38	1.4969	0.54	0.11	0.00	0.782	0.284	0.128	0.000	33.9
2.	Dioxane	2.22	1.4224	0.49	0.37	0.00	0.737	0.312	0.444	0.000	36.0
3.	Chloroform	4.81	1.4459	0.58	0.00	0.44	0.783	0.614	0.071	0.947	39.1
4.	DCM	9.08	1.4242	0.73	0.10	0.13	0.761	0.769	0.178	0.040	40.7
5.	DMF	38.25	1.4305	0.87	0.69	0.00	0.759	0.977	0.613	0.031	43.2
6.	DMSO	47.24	1.4470	1.00	0.76	0.00	0.830	1.000	0.647	0.072	45.1
7.	Acetonitrile	37.50	1.3442	0.75	0.31	0.19	0.645	0.974	0.286	0.044	45.6
8.	Methanol	33.00	1.3288	0.60	0.62	0.93	0.608	0.904	0.545	0.605	55.4

**Table 5:** Values of fitting parameters for naphthalimide derivatives according to Catalan model.

Sample		y_0_ (cm^−1^)	SP	SdP	SB	SA	R^2^
ν_a_	**7**	25,728	−988	−1456	554	−234	0.93
	**8**	26,111	−1353	−1399	328	−336	0.94
ν_f_	**7**	20,204	161	−1095	−539	−138	0.96
	**8**	20,048	552	−1547	−342	86	0.98
Δν	**7**	5525	−1150	−360	1094	96	0.88
	**8**	6072	−1932	117	727	−401	0.82

**Table 6:** Estimated coefficients for multilinear analysis of (ν_a_, ν_f_, Δν) of **7** and **8** as a function of Kamlet–Taft scale.

Sample		ν_0_ (cm^−1^)	π*	β	α	R^2^
ν_a_	**7**	25,985	−2826	736	−973	0.96
	**8**	26,048	−2823	669	−959	0.95
ν_f_	**7**	20,801	−1791	−308	−688	0.92
	**8**	21,090	−2323	−321	−640	0.88
Δν	**7**	5184	−1036	1044	−285	0.85
	**8**	4959	−500	989	−318	0.69

**Table 7:** Regression fit to the solvatochromic parameters of ν_a_, ν_f_, and Δν for **7** and **8** according to Equation (9).

Sample		ν_0_ (cm^−1^)	c_1_	c_2_	c_3_	c_4_	R^2^
ν_a_	**7**	26,851	−1834	−6358	609	−460	0.97
	**8**	27,111	−1815	−7100	518	−498	0.95
ν_f_	**7**	20,040	−1146	741	−309	−66	0.94
	**8**	19,218	−1563	4317	−216	395	0.97
Δν	**7**	6812	−688	−7099	918	−394	0.95
	**8**	7894	−252	−11,418	734	−893	0.95

**Table 8:** Fluorescence lifetimes of naphthalimides **7** and **8** in dioxane/water system.

Sample	Water Content (%)	τ_1_ (ns)/a_1_ (%)	τ_2_ (ns)/a_2_ (%)	τ_av_ (ns)	χ^2^
**7**	0.00	7.24 (100)		7.24	1.00
	0.49	6.58 (100)		6.58	1.00
	0.99	5.81 (100)		5.81	1.00
	2.44	2.48 (56.49)	6.38 (43.51)	5.08	0.81
	4.76	1.46 (62.27)	7.35 (37.73)	5.91	0.85
	20.00	0.43 (27.67)	7.76 (72.33)	7.61	0.96
	39.39	0.43 (11.93)	7.60 (88.07)	7.54	1.00
**8**	0.00	6.66 (100)		6.66	0.99
	0.49	5.83 (100)		5.83	0.97
	0.99	5.22 (100)		5.22	0.98
	2.44	3.50 (100)		3.50	1.00
	4.76	1.68 (79.79)	5.80 (20.21)	3.60	0.68
	20.00	0.46 (59.61)	7.06 (40.30)	6.47	0.75
	39.39	0.26 (34.62)	7.19 (65.38)	7.06	0.89

**Table 9:** Fluorescence lifetimes of naphthalimides in DMF/water mixtures.

Sample	Water Content (%)	τ_1_ (ns)/a_1_ (%)	τ_2_ (ns)/a_2_ (%)	τ_av_ (ns)	χ^2^
**7**	0.00	0.55 (7.93)	8.76 (92.07)	8.71	0.99
	0.99	0.54 (7.49)	8.67 (92.51)	8.62	1.11
	4.76	0.50 (7.44)	8.58 (92.56)	8.53	1.05
	20.00	0.94 (6.84)	8.25 (93.16)	8.18	1.04
	42.86	1.16 (6.59)	7.93 (93.41)	7.86	1.02
	50.00	1.16 (6.04)	7.83 (93.96)	7.77	1.01
**8**	0.00	0.21 (41.46)	7.75 (58.54)	7.61	1.00
	0.99	0.21 (41.03)	7.78 (58.97)	7.64	1.00
	4.76	0.19 (38.67)	7.66 (61.33)	7.54	1.15
	20.00	0.20 (32.23)	7.57 (67.77)	7.47	1.18
	42.86	0.24 (24.03)	7.22 (75.97)	7.14	1.10
	50.00	0.24 (21.26)	7.16 (78.74)	7.09	1.11

**Table 10:** Quenching parameters for naphthalimides in solvent/water mixtures.

Solvent	K_SV_	
	7	8
Dioxane	1.367	2.132
DMF	0.031	0.036
DMSO	0.019	0.018

## Data Availability

The data presented in this study are available on request from the corresponding author.

## Supplementary Materials

The following are available online at https://www.mdpi.com/article/10.3390/ijms23052760/s1.
