# Supplementary material for: Synthesis and Solvent Dependent Fluorescence of Some Piperidine-Substituted Naphthalimide Derivatives and Consequences for Water Sensing
Source: Int J Mol Sci. 2022 Mar 2;23(5):2760. doi: 10.3390/ijms23052760 (PMC8911315; doi:10.3390/ijms23052760)
Supplement: Supplementary file 1 [file ijms-23-02760-s001.zip › ijms-1586411-supplementary.pdf]

Supporting Information

Synthesis and solvent dependent fluorescence of some piperidine-substituted naphthalimide derivatives and consequences for water sensing

Radu Tigoianu [1], Anton Airinei [1,*] Emilian Georgescu [2,3], Alina Nicolescu [1,*], Florentina Georgescu [4], Dragos Lucian Isac [1], Calin Deleanu [1,2], Florin Oancea [5]

[1] Petru Poni Institute of Macromolecular Chemistry, Romanian Academy, Aleea Grigore Ghica Voda 41A, 700487 Iasi, Romania
[2] C. D. Nenitescu Centre of Organic Chemistry, Romanian Academy, Spl. Independentei 202B, 060023 Bucharest, Romania
[3] Research Center, Chimcomplex S.A., St. Uzinei 1, 240050 Ramnicu Valcea, Romania
[4] Enpro Soctech Com srl, Str. Elefterie 51, 050524 Bucharest, Romania
[5] National Research and Development Institute for Chemistry and Petrochemistry – ICECHIM, Spl. Independentei 202B, 060023 Bucharest, Romania
[*] Correspondence: airineia@icmpp.ro; alina@icmpp.ro

List of content

Figure S1. ^{1}H NMR spectrum for compound **7** recorded in DMSO-d_6 at 600 MHz.

Figure S2. ^{13}C NMR spectrum for compound **7** recorded in DMSO-d_6 at 150 MHz.

Figure S3. ^{1}H NMR spectrum for compound **8** recorded in DMSO-d_6 at 600 MHz.

Figure S4. ^{13}C NMR spectrum for compound **8** recorded in DMSO-d_6 at 150 MHz.

Figure S5. ^{1}H-^{15}N HMBC spectrum for compound **8** recorded in DMSO-d_6

Figure S6 Influence of the solvent polarity on the fluorescence quantum yield for **7**.

Figure S7. Influence of the solvent polarity on the fluorescence quantum yield for **8**.

Figure S8 Plots of (**a**) k_r and (**b**) k_{nr} for **7** as a function of solvent polarity parameter

Figure S9. Plots of k_r as a function of solvent polarity parameter for **8**.

Figure S10. Evolution of nonradiative rate constant as a function of $E_T(30)$ for **8**.

Figure S11. Plot of the Stokes shift as a function of solvent polarity parameter according to Eq. (5) for **8**.

Figure S12. Dependence of the Stokes shift of **7** on the solvent polarity function according Eq. (6).

Figure S13. Dependence of the Stokes shift (Δv) on the solvent polarity parameter $E_T(30)$ for **7**.

Figure S14. Plot of Stokes shift of **8** as a function of $E_T(30)$.

Figure S15. Linear relationship between the experimental and predicted absorption maxima using multilinear regression with [f(n), f(ε), β, α] scale for **8**.

Figure S16. Correlation plots between the frequencies estimated using Catalan parameters and the experimental wavenumber values for **7**.

Figure S17. Linear relationship between the experimental and the calculated ν_a for **8** using Kamlet–Taft scale.

Figure S18. Linear relationship between the experimental and predicted Δν using [f(n), f(ε), β, α] scale for **8.**

Figure S19. Profile of the electronic absorption spectra versus water content of **7** in dioxane solution.

Figure S20. Fluorescence spectra of **7** in DMF as a function of water percentage (λ_{ex} = 417 nm, 342 nm).

Figure S21. Emission spectra of **8** in DMSO adding different water amounts (λ_{ex} = 420 nm).

Figure S22. 3D Fluorescence spectra of **7** in different solvents (a) dioxane and (b) DMF.

Figure S23. Fluorescence decay profile of **7** in dioxane in the presence of increasing water content.

Figure S24. Fluorescence decay curves of **7** in DMF with increasing water content.

Figure S25. Time resolved fluorescence decay of **8** in DMF with different water levels.

Figure S26. Emission spectra of **7** in chloroform with different dichloromethane amounts (λ_{ex} = 417 nm).

Figure S27. Fluorescence spectra of **8** in chloroform with different dichloromethane fractions (λ_{ex} = 417 nm).

Figure S28. Stern–Volmer plots for changes in emission intensity of **7** in DMSO as a function of the water level.

Figure S29. Stern–Volmer plots for changes in emission intensity of **7** in DMF as a function of the water level.

Figure S30. S-V plot for **8** in dioxane with different contents of water.

Figure S31. S-V plots for fluorescence quenching of **8** in DMF adding different water levels.

Figure S32. S-V plots for fluorescence quenching of **8** in DMSO adding different water levels.

Figure S33. Emission spectra of **7** in DMSO at different temperatures.

Figure S34. Plot of the fluorescence intensity of **8** *versus* water content in dioxane.

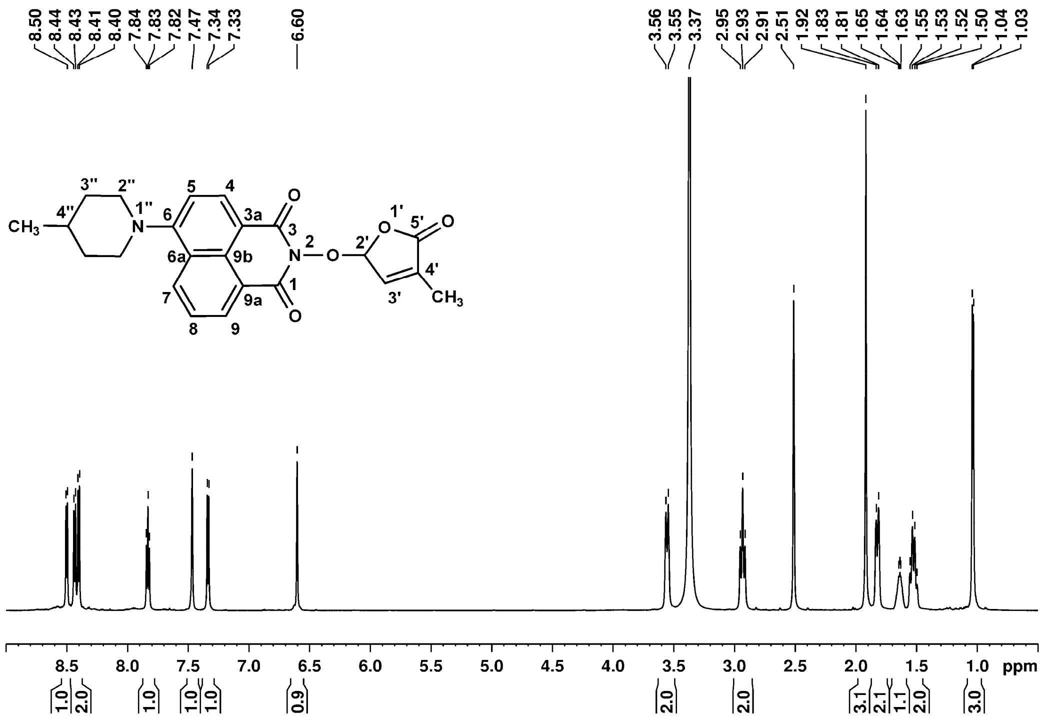

Figure S1. ^{1}H NMR spectrum for compound **7** recorded in DMSO-d6 at 600 MHz.

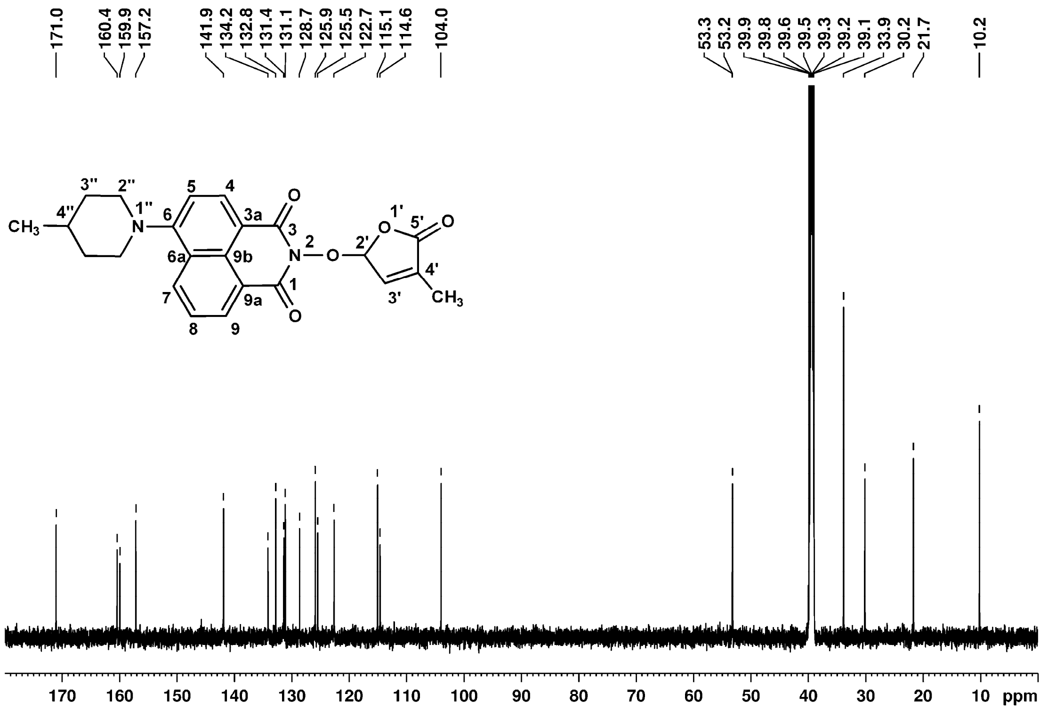

Figure S2. ^{13}C NMR spectrum for compound **7** recorded in DMSO-d_6 at 150 MHz.

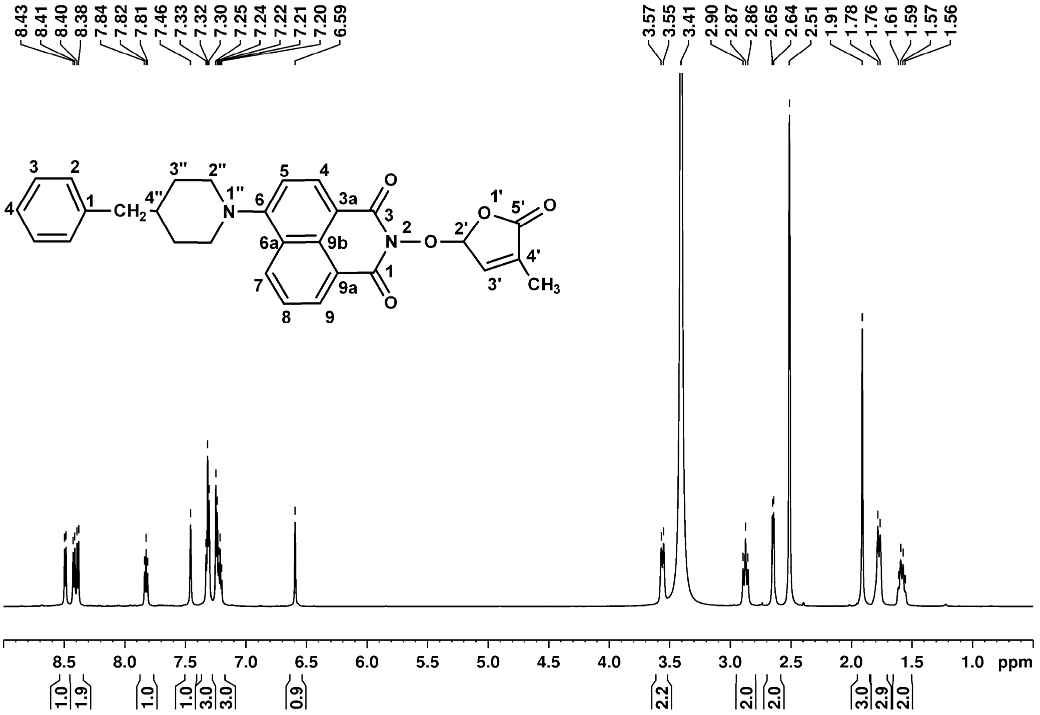

Figure S3. ^1H NMR spectrum for compound **8** recorded in DMSO-d_6 at 600 MHz.

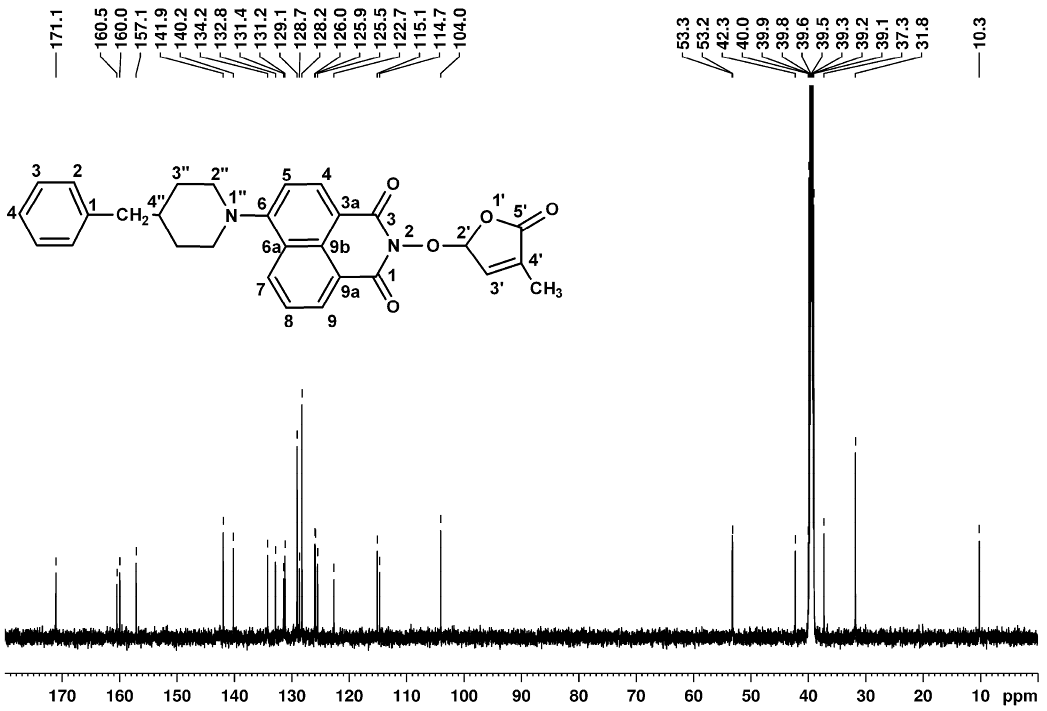

Figure S4. ^{13}C NMR spectrum for compound **8** recorded in DMSO-d6 at 150 MHz.

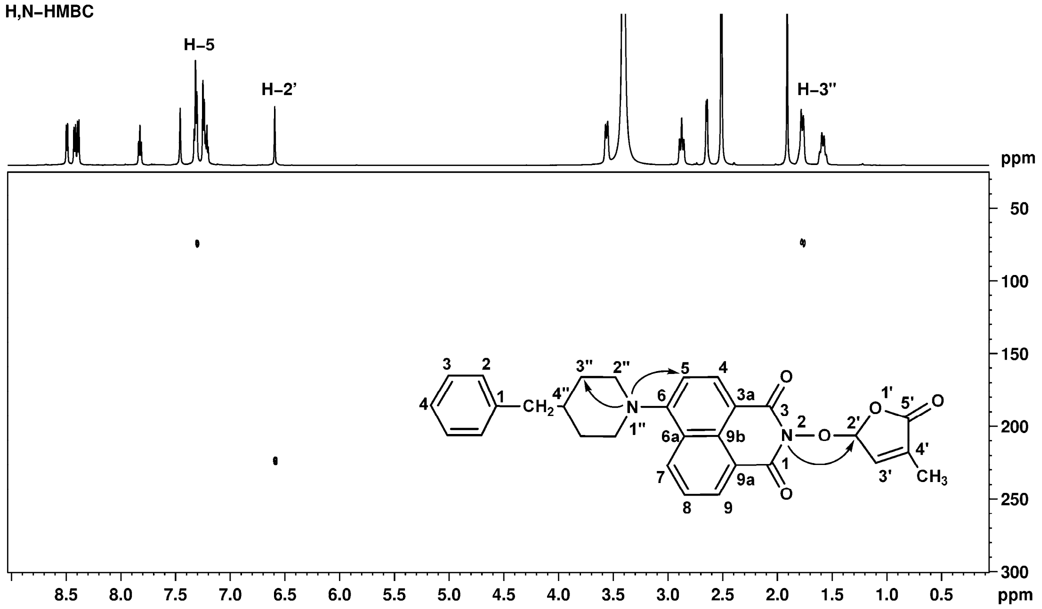

Figure S5. ^{1}H-^{15}N HMBC spectrum for compound **8** recorded in DMSO-d6.

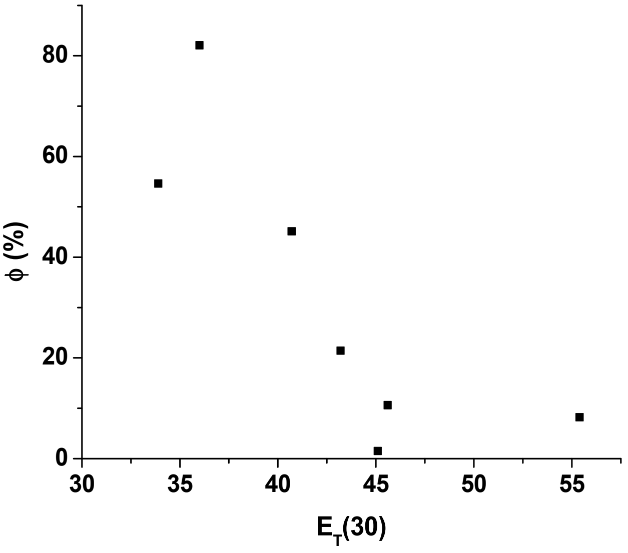

Figure S6. Influence of the solvent polarity on the fluorescence quantum yield for **7**.

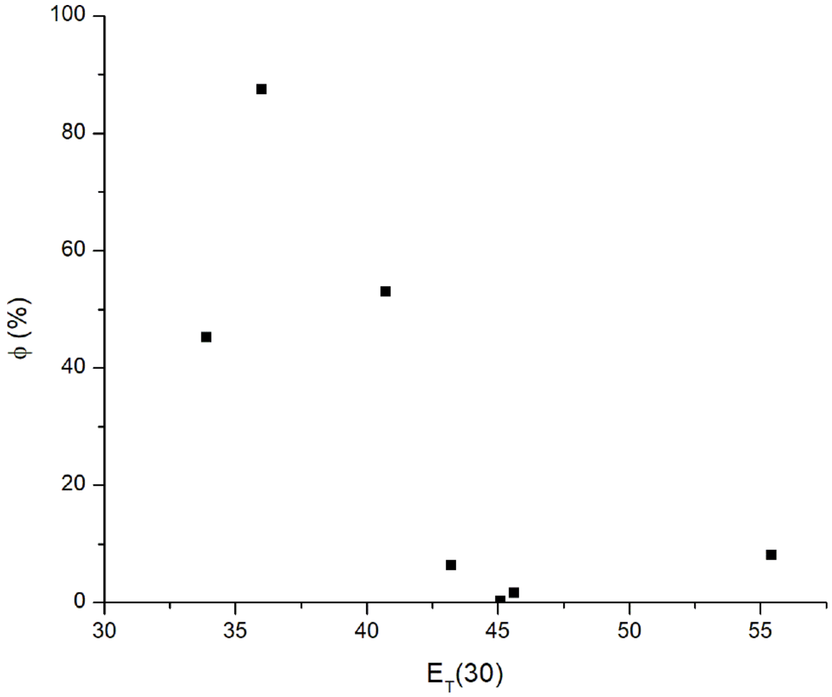

Figure S7. Influence of the solvent polarity on the fluorescence quantum yield for **8**.

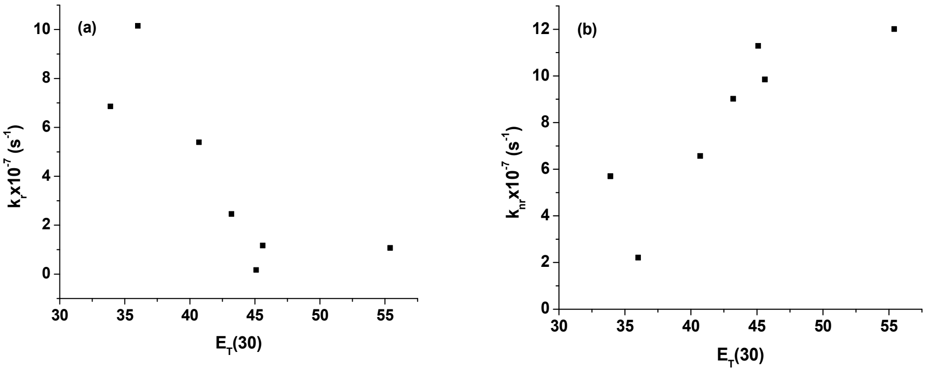

Figure S8. Plots of (**a**) k_r and (**b**) k_{nr} for **7** as a function of solvent polarity parameter $E_T(30)$.

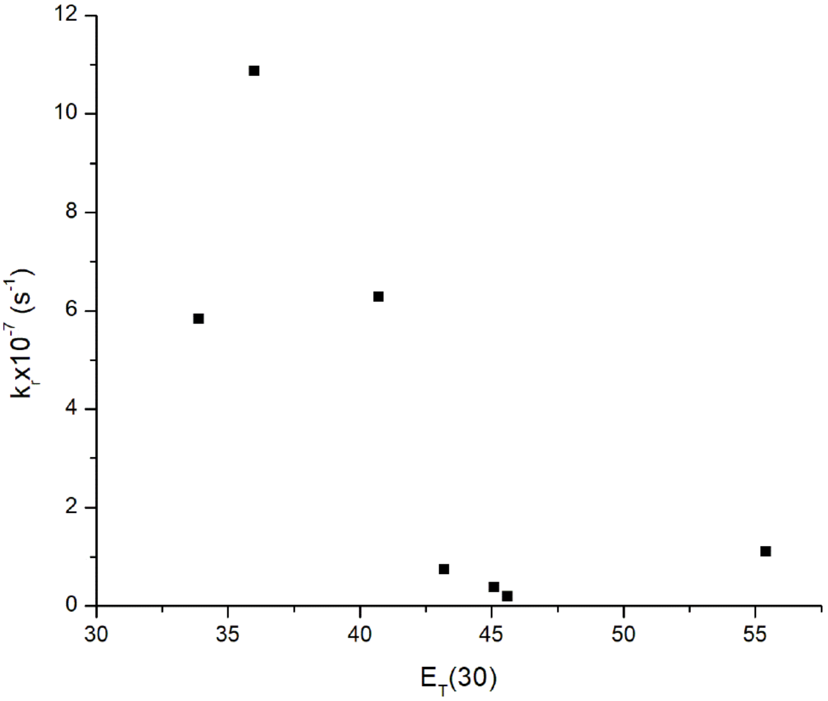

Figure S9. Plot of k_r as a function of solvent polarity parameter for **8**.

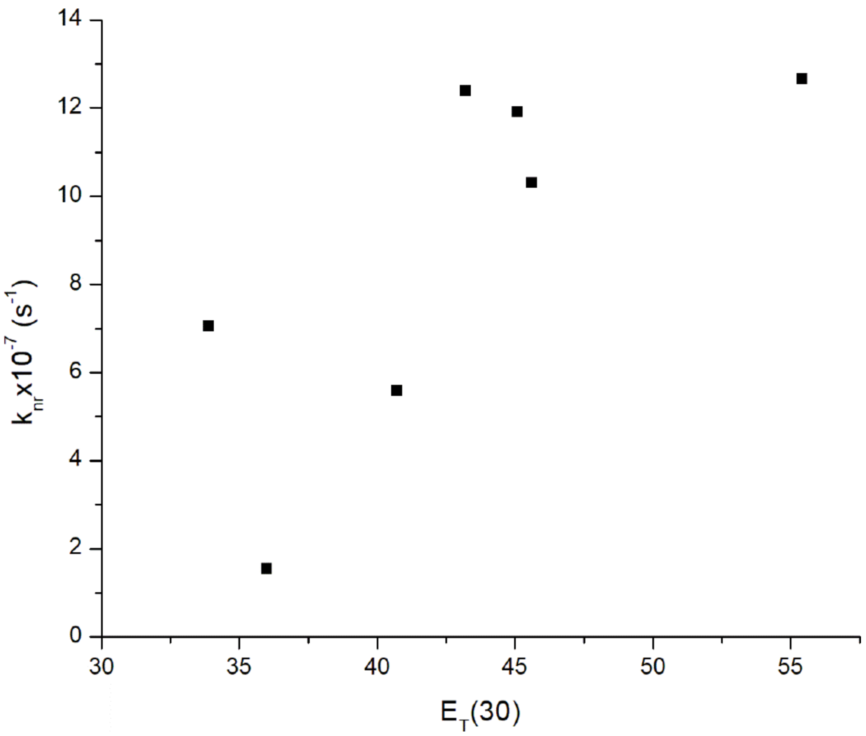

Figure S10. Evolution of nonradiative rate constant as a function of $E_T(30)$ for **8**.

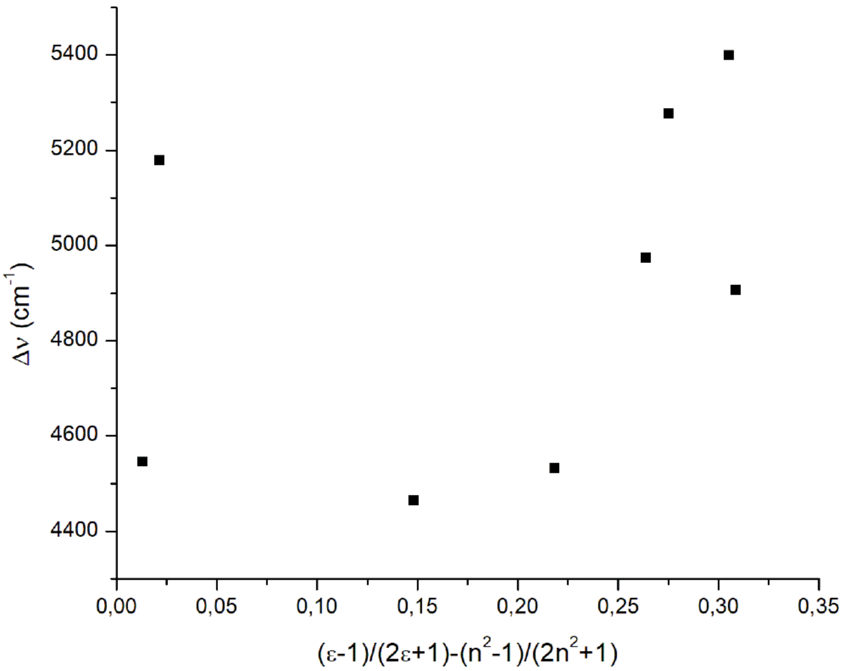

Figure S11. Plot of the Stokes shift as a function of solvent polarity parameter according to Eq. (5) for **8**.

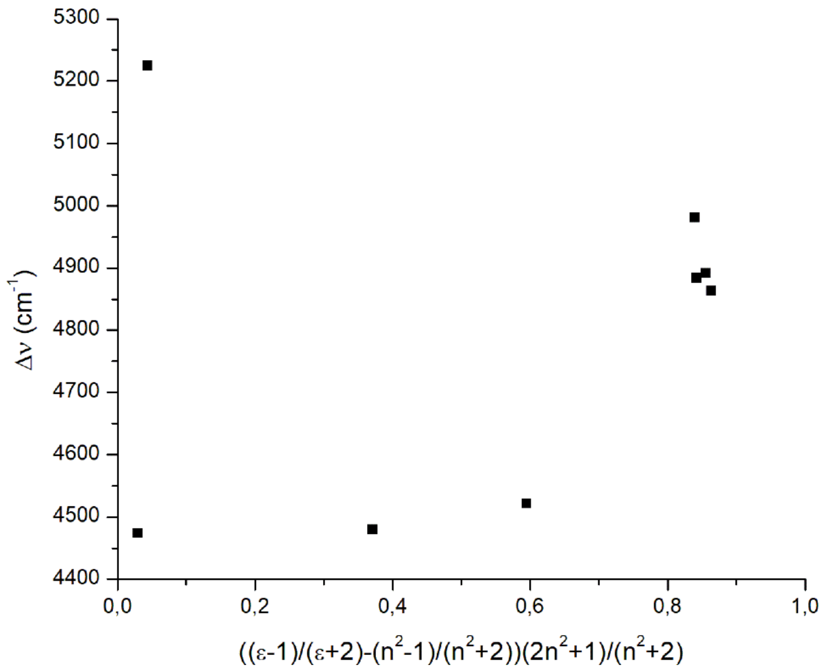

Figure S12. Dependence of the Stokes shift of **7** on the solvent polarity function according to Eq. (6).

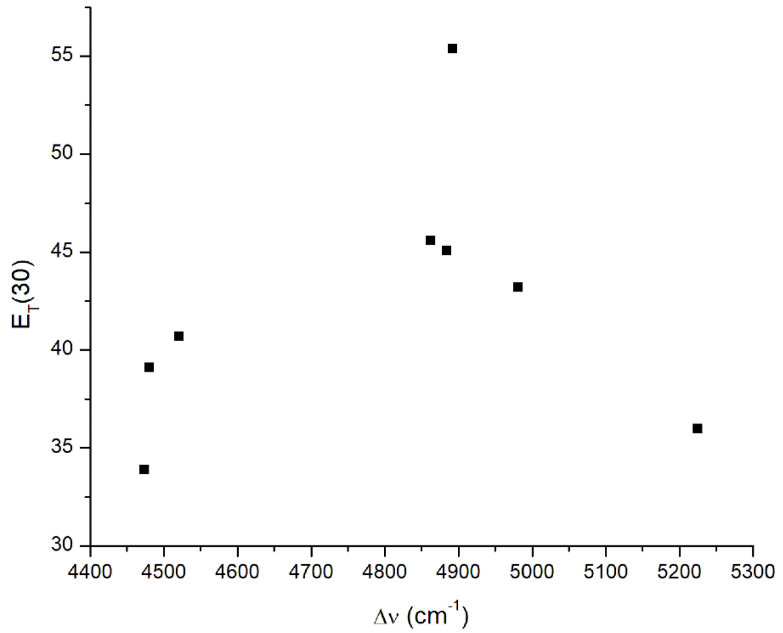

Figure S13. Dependence of the Stokes shift (Δν) on the solvent polarity parameter $E_T(30)$ for 7.

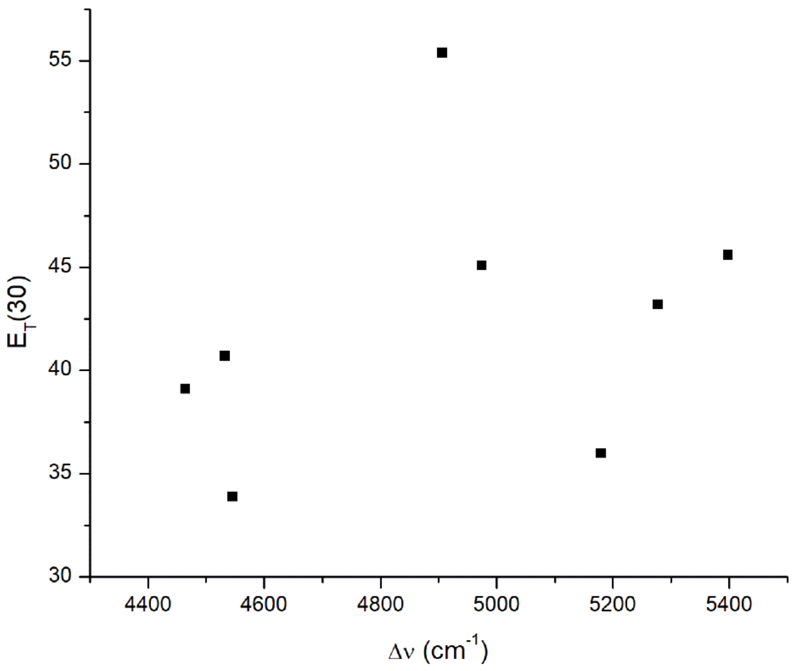

Figure S14. Plot of Stokes shift of **8** as a function of $E_T(30)$.

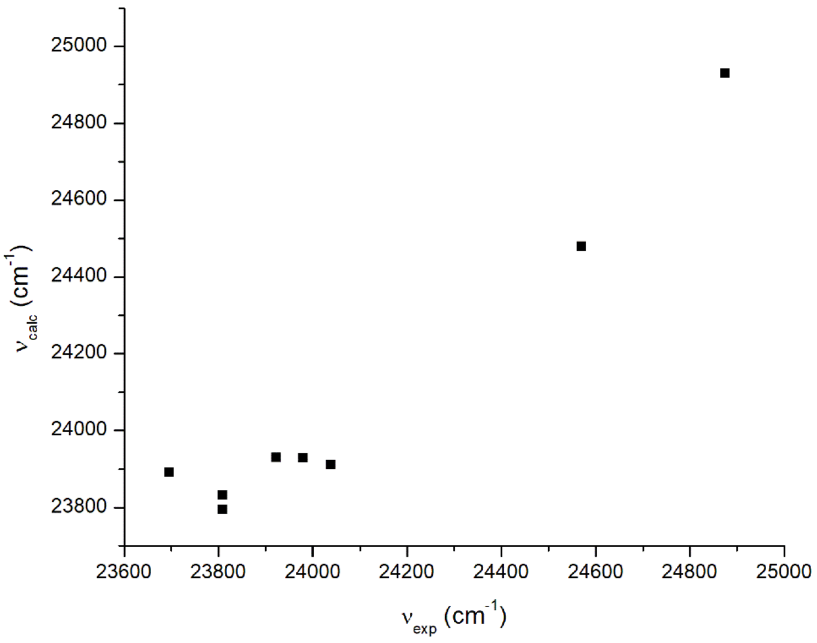

Figure S15. Linear relationship between the experimental and predicted absorption maxima using multilinear regression with [f(n), f(ε), β, α] scale for **8**.

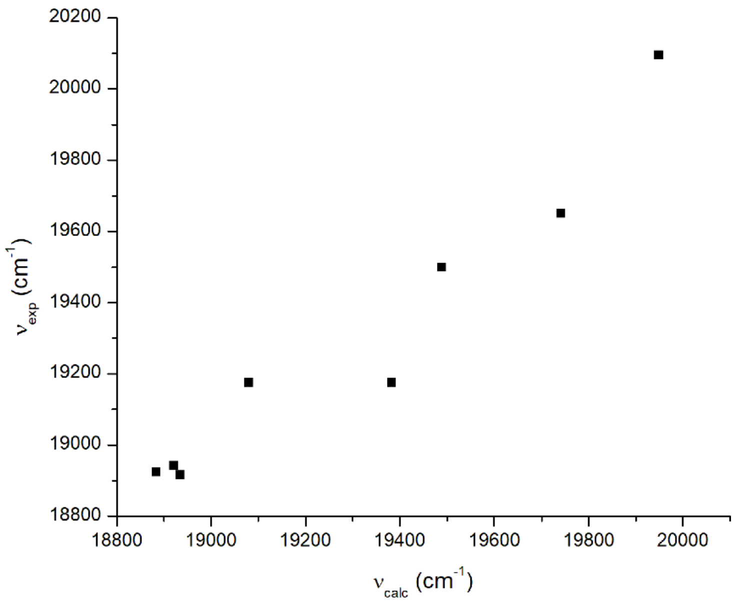

Figure S16. Correlation plots between the frequencies estimated using Catalan parameters and the experimental wavenumber values for **7**.

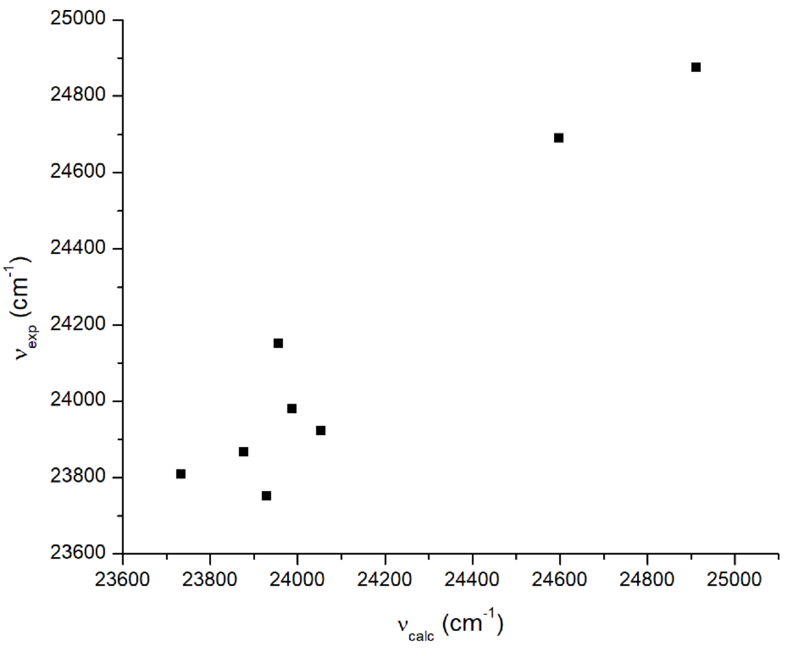

Figure S17. Linear relationship between the experimental and the calculated ν_a for **8** using Kamlet–Taft scale.

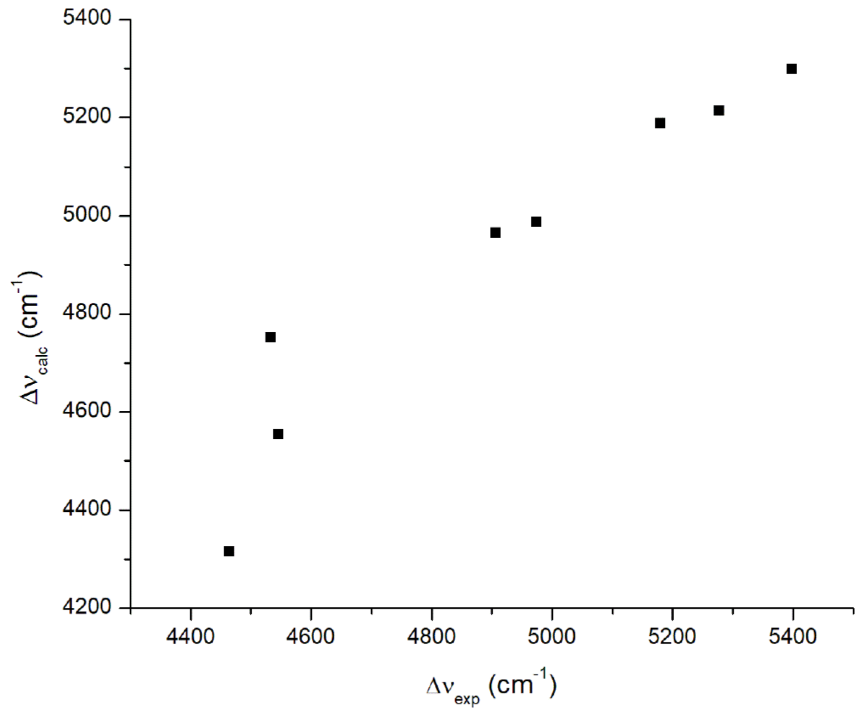

Figure S18. Linear relationship between the experimental and predicted Δν using [f(n), f(ε), β, α] scale for **8**.

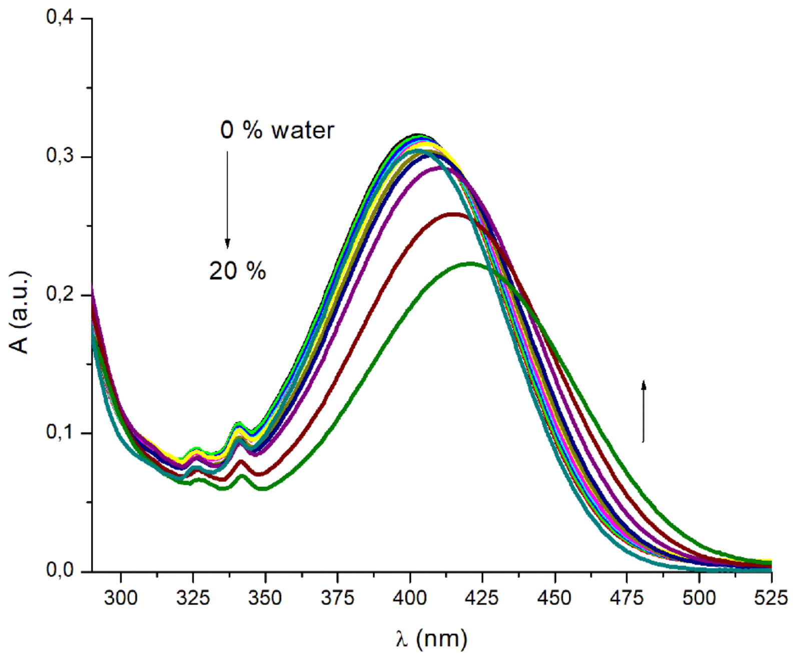

Figure S19. Profile of the electronic absorption spectra versus water content of **7** in dioxane solution.

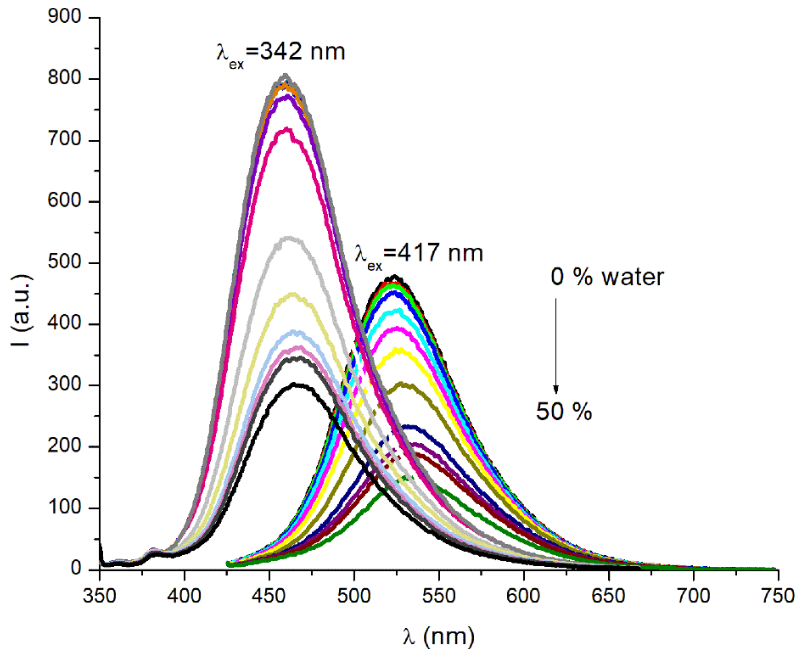

Figure S20. Fluorescence spectra of **7** in DMF as a function of water percentage (λ_{ex} = 417 nm, 342 nm).

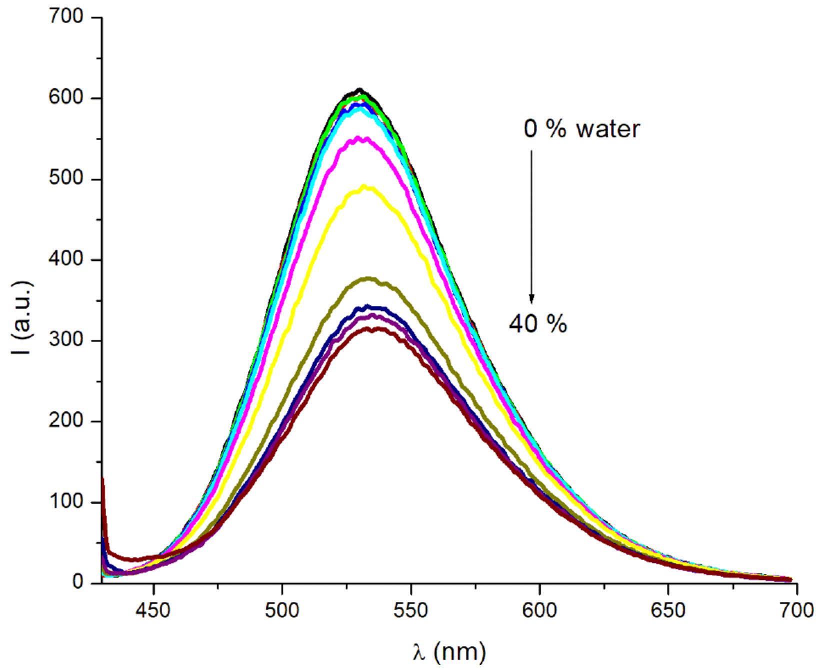

Figure S21. Emission spectra of **8** in DMSO adding different water amounts (λ_{ex} = 420 nm).

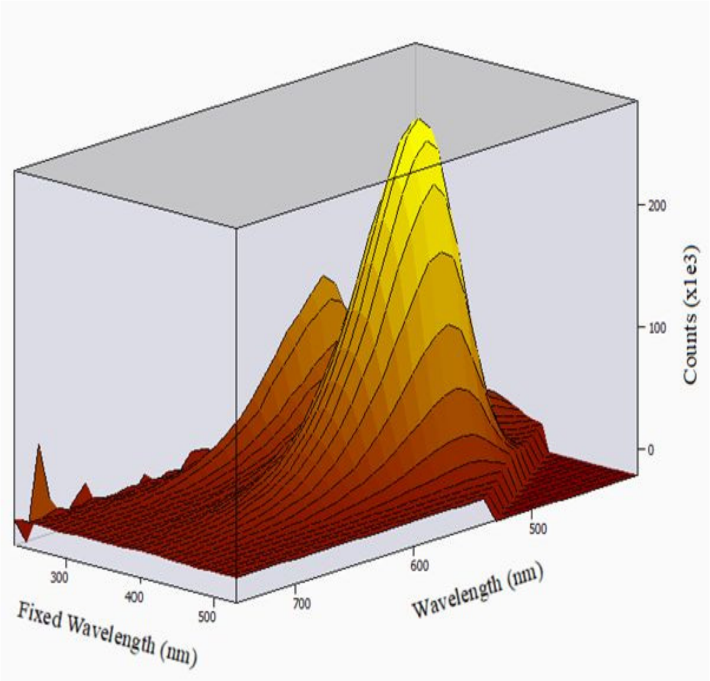

Figure S22a. 3D Fluorescence spectra of **7** in dioxane.

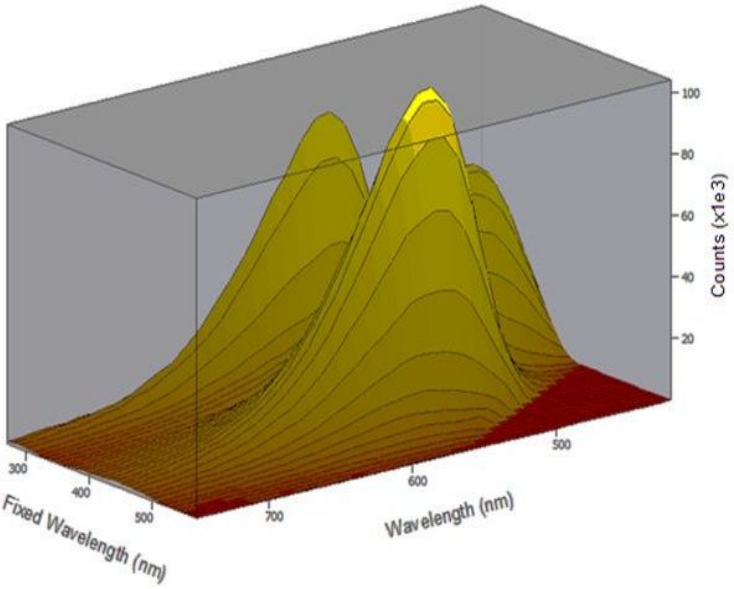

Figure S22b. 3D Fluorescence spectra of **7** in DMF.

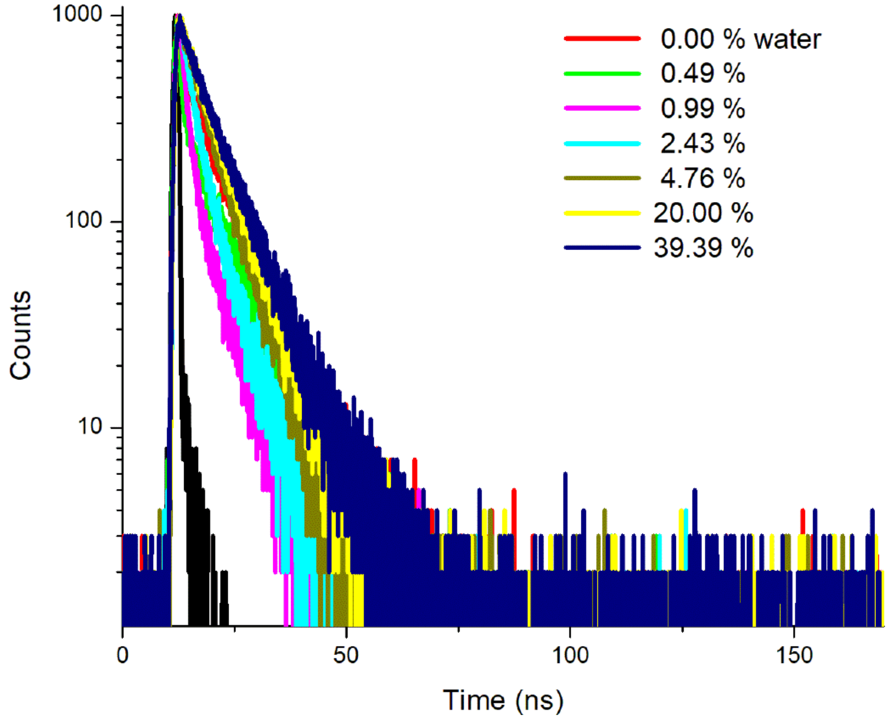

Figure S23. Fluorescence decay profile of **7** in dioxane in the presence of increasing water content.

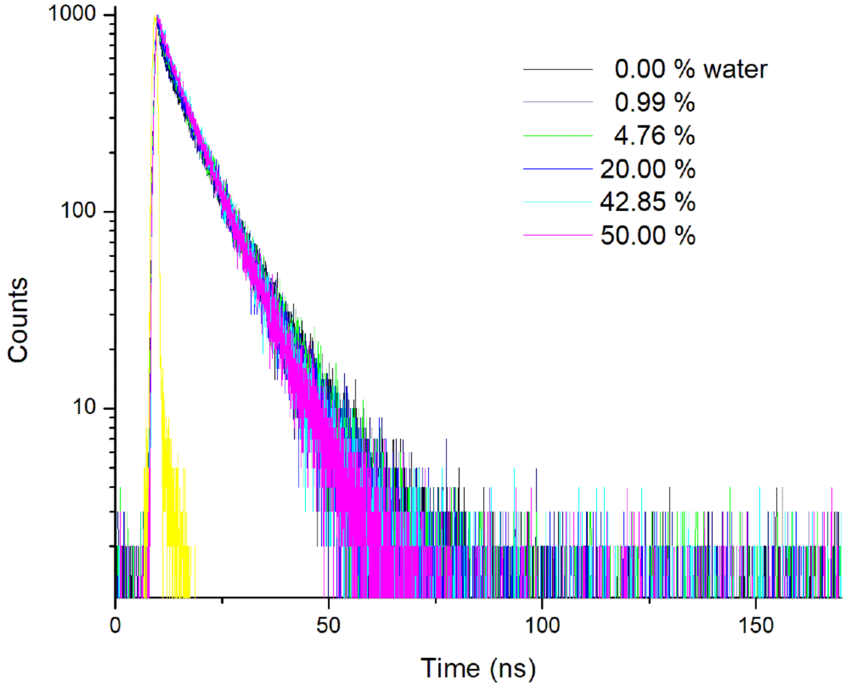

Figure S24. Fluorescence decay curves of **7** in DMF with increasing water content.

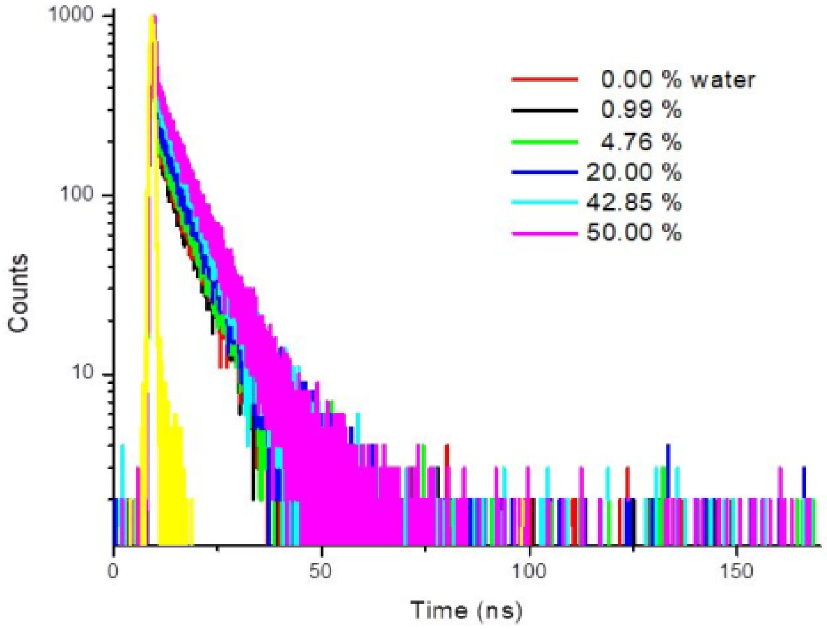

Figure S25. Time resolved fluorescence decay of **8** in DMF with different water levels.

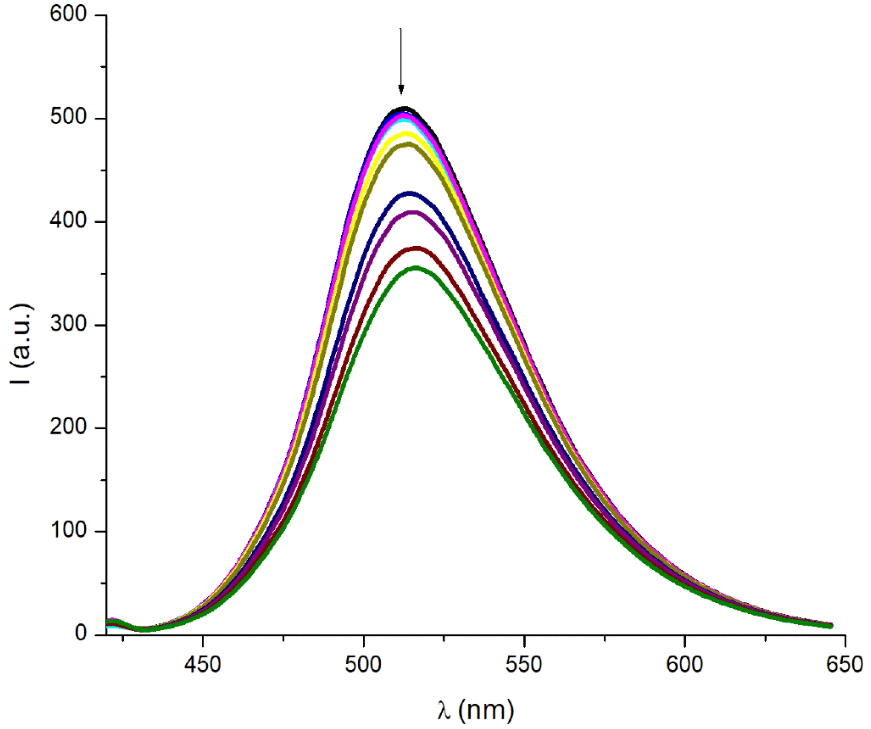

Figure S26. Emission spectra of **7** in chloroform with different dichloromethane amounts (λ_{ex} = 417 nm).

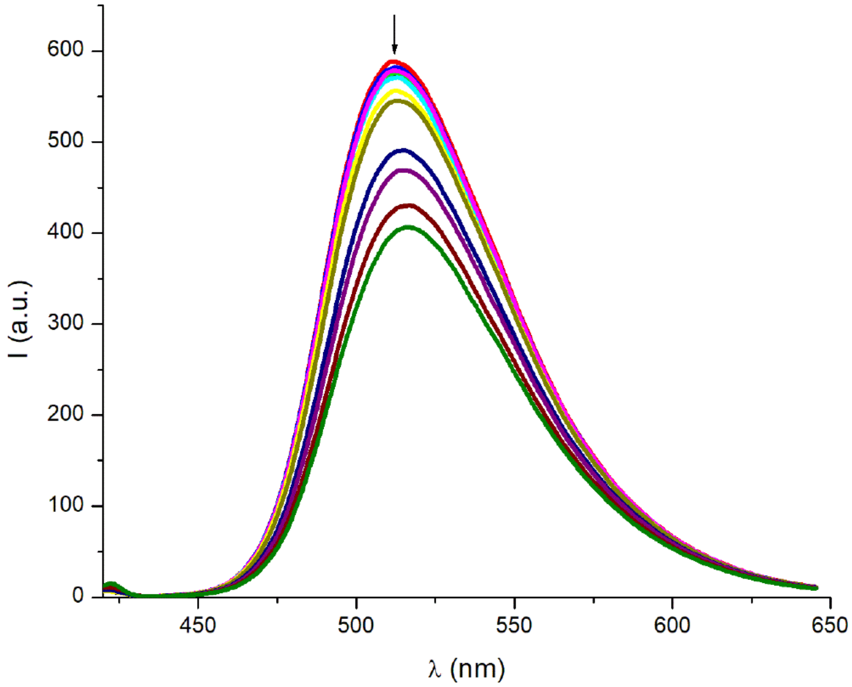

Figure S27. Fluorescence spectra of **8** in chloroform with different dichloromethane fractions (λ_{ex} = 417 nm).

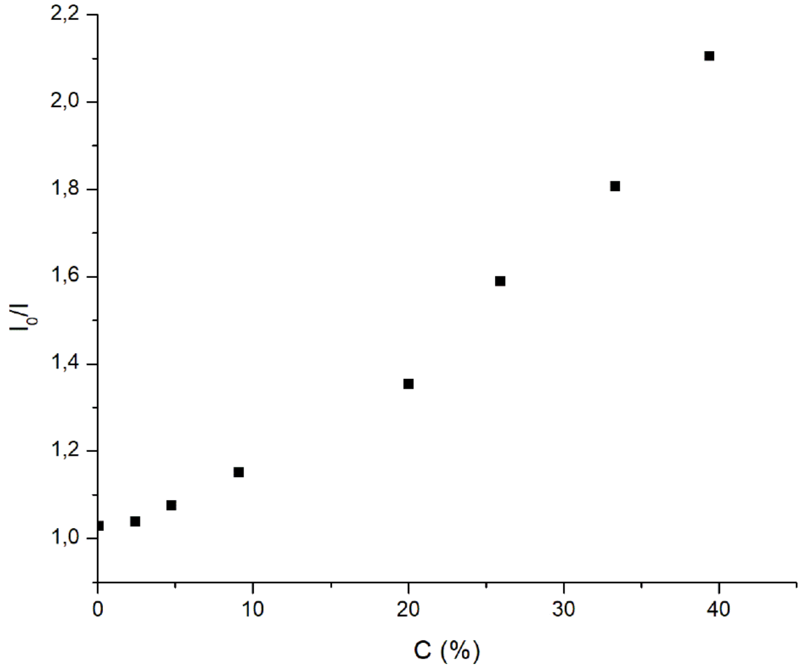

Figure S28. Stern–Volmer plots for changes in emission intensity of **7** in DMSO as a function of the water level.

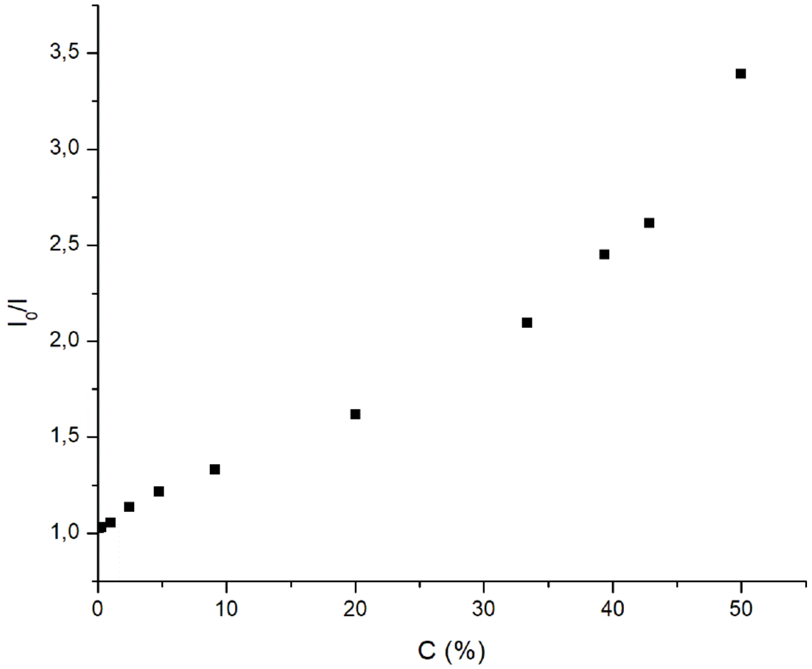

Figure S29. Stern–Volmer plots for changes in emission intensity of **7** in DMF as a function of the water level.

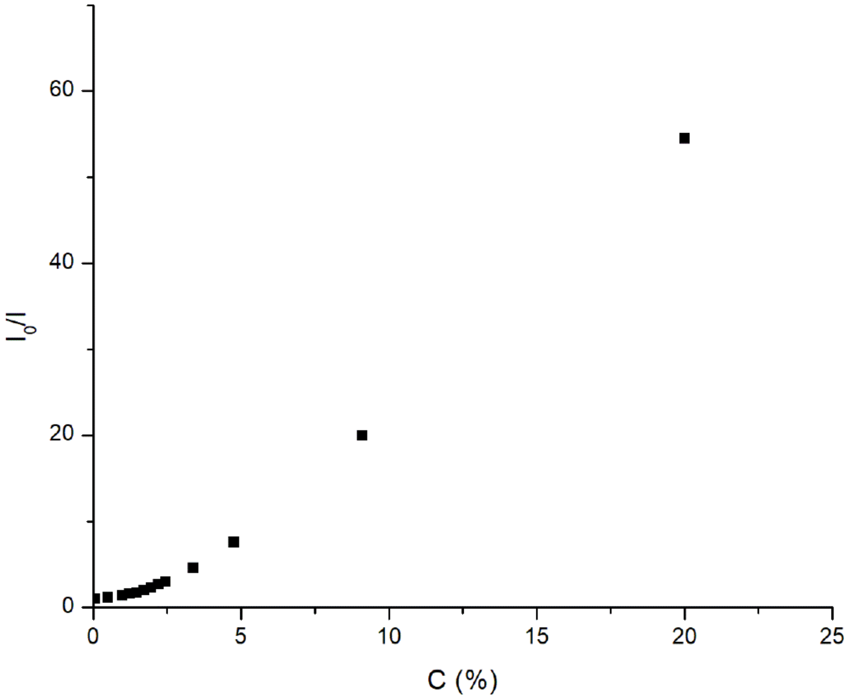

Figure S30. S-V plot for **8** in dioxane with different contents of water.

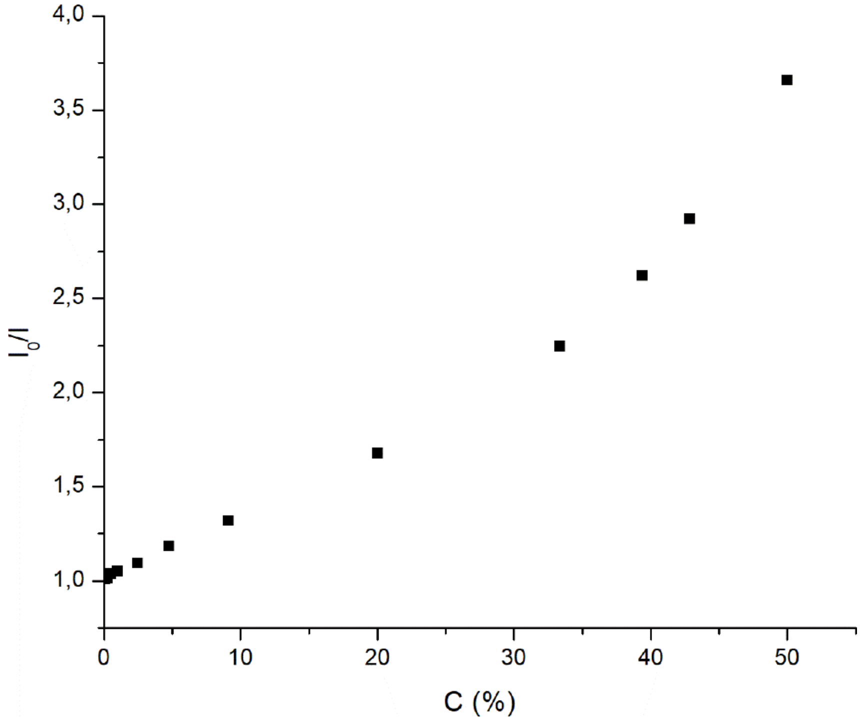

Figure S31. S-V plots for fluorescence quenching of **8** in DMF adding different water levels.

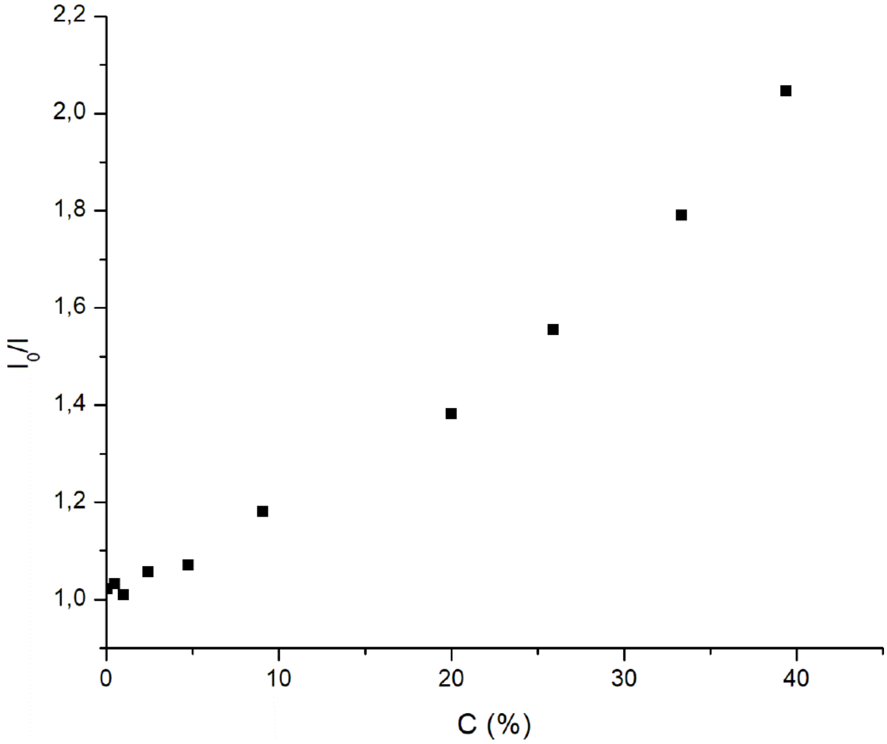

Figure S32. S-V plots for fluorescence quenching of **8** in DMSO adding different water levels.

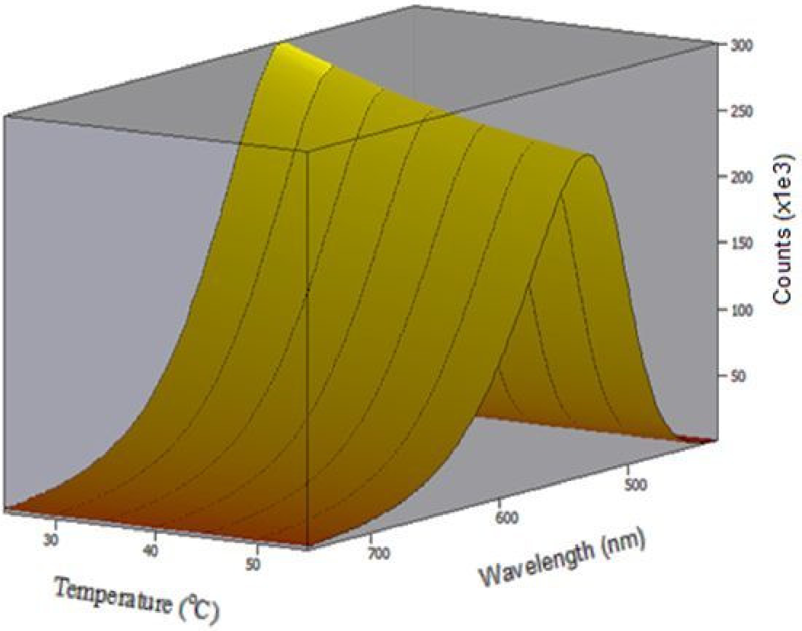

Figure S33. Emission spectra of **7** in DMSO at different temperatures.

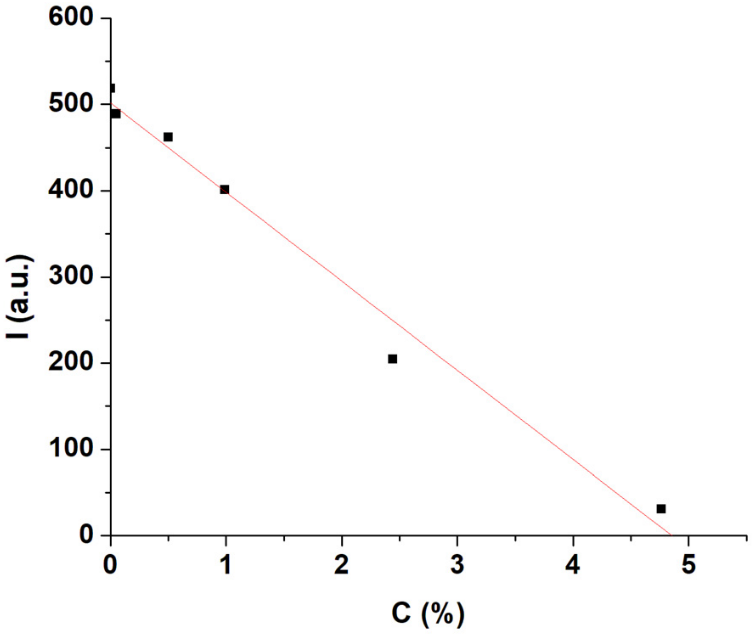

Figure S34. Plot of the fluorescence intensity of **8** versus water content in dioxane.
